# Controlling Heterogeneous Catalysis With Subsurface Oxygen

**DOI:** 10.1002/anie.202524699

**Published:** 2026-02-17

**Authors:** Arved C. Dorst, Zhikai Jiang, Maxwell Gillum, Rasika E. A. Dissanayake, Bin Jiang, Daniel R. Killelea, Tim Schäfer

**Affiliations:** ^1^ Institute of Physical Chemistry University of Göttingen Göttingen Germany; ^2^ Max‐Planck‐Institute for Multidisciplinary Sciences Göttingen Germany; ^3^ State Key Laboratory of Precision and Intelligent Chemistry Department of Chemical Physics University of Science & Technology of China Hefei Anhui P. R. China; ^4^ Department of Chemistry & Biochemistry Loyola University Chicago Chicago Illinois USA

**Keywords:** CO oxidation on Rh surfaces, DFT calculations, ion imaging, molecular beam surface scattering, subsurface oxygen

## Abstract

Rhodium surfaces play a crucial role in heterogeneous catalysis, driving extensive research on their reactivity. In particular, CO oxidation is of great interest, where different oxygen species at the surface can influence catalytic activity. Under certain conditions, rhodium can also host sub‐surface oxygen species, further affecting reaction dynamics. In this work, we combine molecular beam surface scattering, ion imaging, and ultra‐high vacuum techniques to investigate the impact of subsurface oxygen on CO oxidation on single‐crystal Rh surfaces. When oxidizing CO at the (2 × 1)‐O adlayer without subsurface oxygen, we observe hyperthermal velocity distributions of desorbing CO_2_, indicating significant energy release along the translational coordinate directly from the transition state. In contrast, subsurface oxygen induces thermal velocity distributions. DFT calculations indicate the formation of a chemisorption state in presence of subsurface oxygen that is energetically favored and transiently traps product CO_2_ long enough for thermalization.

## Graphical abstract

During CO oxidation on rhodium surfaces, the reaction dynamics are markedly altered in the presence of subsurface oxygen. Subsurface oxygen stabilizes the nascent physisorbed CO_2_ formed after the surface reaction, allowing it to become transiently trapped and thermalize with the surface. This results in substantially modified post‐transition‐state dynamics.

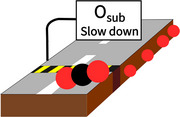

## 1 Introduction

Heterogeneously catalyzed oxidation reactions over late transition metal surfaces are essential to modern industrial chemistry and have found widespread applications in many key areas of the modern chemical infrastructure [1, 2, 3, 4, 5, 6]. Despite their broad relevance, these reactions continue to resist a unified, atomic‐level description of how energy flows during catalysis and how specific surface sites dictate mechanistic pathways [7, 8, 9, 10, 11]. To address this challenge, modern surface science has focused on isolating and interrogating the elementary steps of surface reactivity under well‐controlled ultra‐high‐vacuum conditions using well‐defined single‐crystal substrates [1, 3, 4, 12]. Insights gained from such model studies provide the foundation for constructing a more comprehensive picture of catalytic surface chemistry [13].

The greater the control an experiment provides over its conditions, the more reliably clear conclusions can be drawn from its outcomes. When probing surface characteristics under catalytic conditions, achieving such clarity requires sophisticated experimental approaches that minimize perturbation of the system and operate in tandem with robust theoretical models to enable meaningful interpretation [14, 15, 16, 17, 18, 19, 20, 21, 22, 23, 24, 25]. X‐ray and electron‐based techniques, although powerful, typically demand intensities at which perturbation or even damage of the probed volume becomes a significant concern. Reducing the intensity to mitigate these effects necessitates extensive signal averaging, which in turn compromises temporal resolution. Theoretical models face comparable challenges due to the intrinsic complexity of heterogeneously catalyzed systems. Even the simplest surface defects require tracking tens of metal atoms and their associated electrons. Moreover, reactants may occupy multiple adsorption sites whose energetics are strongly coupled to neighboring environments, further complicating predictive descriptions. For these reasons—along with others not discussed here and those yet to be uncovered—heterogeneous catalysis has remained a challenging and vibrant area of research for decades, and it promises to continue stimulating intensive investigation for many years to come [10, 26, 27, 28, 29].

For instance, additional non‐invasive control over the experimental conditions can be achieved by probing not only the surface itself but also the desorbing reaction products. This approach provides valuable mechanistic insight, as these products carry information about the interfacial reaction—including the population of internal degrees of freedom. Laser spectroscopy offers a powerful means of accessing such state‐selective information on surface reaction products after desorption [15, 30, 31, 32, 33, 34, 35, 36]. Such data enable detailed conclusions about surface reaction mechanisms and kinetics. This level of control is even enhanced with a recently developed technique in Göttingen that combines molecular‐beam surface scattering with ion imaging after laser ionization, providing unprecedented insight into surface reaction dynamics and kinetics. It yields velocity distributions of desorbing reaction products near the surface and enables the determination of total product flux after initiating surface reactions via adsorption from pulsed molecular beams. The pulsed beam also allows extraction of rate constants on the millisecond time scale, set by its repetition rate (velocity‐resolved kinetics, VRK). Since its first application in 2018 to CO oxidation on Pt surfaces [37], this method has been applied to a variety of reactions, including H_2_ dissociation on Cu(111) [38], H_2_ oxidation on Pd [39], styrene oxidation on Ag(111) [40], formic acid decomposition on Pd [41, 42], N_2_O decomposition on Pd(110), [43] oxygen recombination on Ag(111) [44], and NH_3_ diffusion on Pt(111) [17]. In close interplay with theory, these experiments provide mechanistic insight by allowing the comparison between measured kinetics and dynamical fingerprints—such as velocity distributions of desorbing products—with theoretical predictions.

In this work, we apply this technique to investigate the influence of subsurface oxygen (O_sub_) on surface reactivity. O_sub_ can significantly influence reactivity in heterogeneous catalysis and is often found at Pt, Pd, Ru, and Rh surfaces [20, 45, 46, 47, 48, 49, 50, 51, 52, 53, 54, 55, 56, 57, 58, 59, 60]. It refers to the incorporation of oxygen atoms beneath the topmost layers of the lattice, typically forming under strongly oxidizing conditions. When O_sub_ is abundant, total oxygen coverages can exceed one monolayer [46]. In such cases, the surface typically consists of an oxidized top layer, with the remaining oxygen residing in subsurface sites. Its proximity to the surface can influence surface chemistry in several ways. On one hand, it can act as a reservoir, diffusing to the surface to replenish oxygen and regenerate active sites. On the other hand, it can modify the electronic and geometric structure of the surface, thereby altering the potential energy surface (PES). Through such effects, subsurface oxygen can indirectly impact surface reactivity.

In general, formation, stability, and influence of subsurface oxygen on surface structures are complex and vary significantly from metal to metal [2]. As a result, different experimental procedures are required to prepare O_sub_ under well‐controlled UHV conditions. For Rh surfaces, exposure to molecular oxygen readily forms dissociated adsorbed oxygen (O_ad_) with a well‐defined structure and composition, saturating at a coverage (θ_O_) of 0.5 ML in a (2 × 1)‐O adlayer [2]. Under these conditions, the rate of subsurface oxygen formation is negligible [61]. High pressures of molecular oxygen are required to form high‐oxygen phases and O_sub_ [62, 63]. In UHV, subsurface oxygen formation can be enhanced by using atomic oxygen (AO). Exposure of Rh surfaces to AO at room temperature produces high‐oxygen‐content phases along with O_sub_, while at elevated temperatures, AO exposure leads to a coexistence of the (2 × 1)‐O adlayer, single‐layer RhO_2_, and O_sub_. [62, 63, 64] While the formation and structure of oxygen‐covered Rh surfaces and those containing subsurface oxygen have been extensively studied, less is known about the details of their chemical reactivity.

In general, rhodium surfaces are highly catalytically active, playing a crucial role in heterogeneous oxidation reactions such as the partial oxidation of methane to syngas [65, 66, 67, 68]. Consequently, reactions on Rh have attracted considerable attention, making it a widely used model system for studying surface‐mediated oxidation. In particular, CO oxidation has been extensively studied, with attention to the roles of different surface oxygen species—adsorbed oxygen (O_ad_), bulk oxide (Rh_2_O_3_), and surface oxide (RhO_2_)—in the reaction [16, 69, 70, 71, 72]. The influence of O_sub_ on chemical reactivity is more challenging to probe experimentally, as it demands a high level of control over experimental conditions [73].

In a recent study, we used VMI techniques to investigate velocity distributions of recombinatively desorbing oxygen from subsurface states in Rh(111) surfaces [56]. In the presented work herein, we extend this approach to examine the reactivity of Rh surfaces with subsurface oxygen, using the same methods to measure velocity distributions of CO_2_ produced by CO oxidation on Rh surfaces with and without subsurface oxygen. Coupling these experiments with DFT calculations enables us to address a key question: Does subsurface oxygen react directly with CO at the surface, or does it indirectly modify the potential energy surface (PES) and thereby alter the reaction pathway? Our results provide strong evidence for the latter—subsurface oxygen profoundly reshapes the PES, opening reaction pathways inaccessible in its absence.

## 2 Results and Discussion

### 2.1 Production of Subsurface Oxygen at Rh(111) and Rh(332)

**FIGURE 1:**
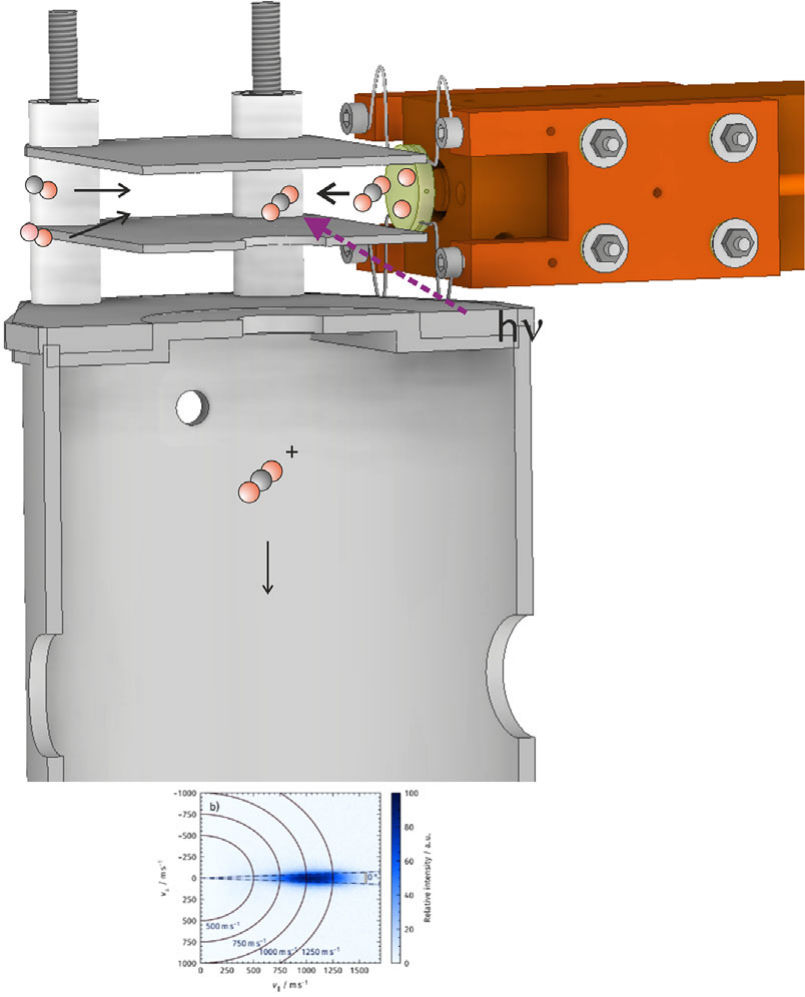
Velocity map imaging (VMI) detector positioned in front of the surface captures desorbing CO_2_ reaction products following CO oxidation on an oxidized Rh surface. CO and O_2_ are introduced via pulsed molecular beams. For subsurface oxygen generation, a hot Ir filament can be placed in front of the surface while backfilling the chamber with O_2_ and producing atomic oxygen (AO). Desorbing products are non‐resonantly ionized using a 35 fs, 800 nm femtosecond laser pulse and accelerated toward a microchannel plate MCP/phosphor screen detector. Time‐of‐flight techniques enable mass discrimination. From the ion impact positions on the phosphor screen, velocity distributions of the desorbing reaction products are reconstructed. Further details of the experimental setup are provided in the Supporting Information.

We employ molecular beam surface scattering combined with velocity map imaging (VMI) to characterize the velocity distributions of the CO_2_ reaction product from CO oxidation on oxidized Rh surfaces, both with and without subsurface oxygen (O_sub_). Details of the experimental setup are provided in the Supporting Information and have been given in previous publications [44, 56]. Briefly, a UHV surface science chamber is connected to two pulsed molecular beam nozzles via differential pumping, maintaining UHV conditions in the scattering chamber during operation. The nozzles deliver pulsed beams with typical durations of 100 µs. One nozzle introduces O_2_ (500 m/s) to oxidize the Rh surfaces, while the other supplies CO (590 m/s). The repetition rates of the nozzles can be independently adjusted. The CO_2_ product is ionized using a femtosecond laser (35 fs, 800 nm, Solstice, Spectra‐Physics) and imaged using a VMI detector, as shown in Figure 1.

We investigate CO oxidation on a split bifaceted Rh crystal that exposes both (111) and (332) surface orientations and is prepared using standard procedures [74, 75, 76]. The disk‐shaped Rh crystal has a diameter of 10 mm, allowing for surface switching by translating the crystal by 5 mm.

**FIGURE 2:**
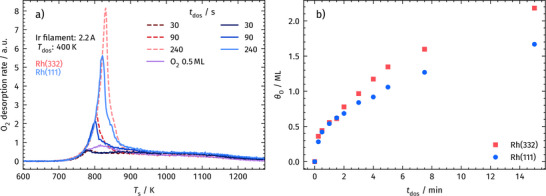
Panel a): TPD spectra of recombinatively desorbing O_2_ following atomic oxygen (AO) exposure on a bifaceted Rh crystal. AO dosage time (𝑡_do__s_) controls the amount of oxygen deposited. Spectra from Rh(332) and Rh(111) are shown as dashed and solid lines, respectively. A sharp desorption peak near 800 K corresponds to O_2_ from subsurface oxygen (O_sub_), followed by broad recombinative desorption of surface oxygen up to 1275 K. For reference, a TPD trace after pure O_2_ exposure (purple) shows no O_sub_ contribution. Panel b): Integrated TPD signals of O_2_ desorption as a function of AO dosage time (𝑡_do__s_). The integrals are referenced to O_2_ dosing data corresponding to 0.5 ML coverage. Once surface saturation is reached, subsurface oxygen (O_sub_) uptake begins. For identical 𝑡_o__s_, the (332) surface incorporates more O_sub_ than the (111) surface.

To obtain a surface containing exclusively chemisorbed oxygen (i.e., no subsurface O), we expose the crystal to the molecular‐oxygen beam for 5 min at 100 Hz, which reliably saturates the adlayer. To prepare a fully covered oxygen surface containing O_sub_, we instead expose the crystal to atomic oxygen generated by backfilling the chamber with O_2_ while positioning a hot Ir filament (2.2 A, ∼1500 K) in front of the surface [77, 78]. The formation of subsurface oxygen is characterized by a sharp desorption peak near 800 K in temperature‐programmed desorption (TPD) experiments, as shown in Figure 2.

Figure 2a shows the O_2_ TPD spectra for varying AO exposures on both the Rh(111) and Rh(332) facet. The surface is resistively heated with a constant temperature ramp while monitoring *m/z* = 32 (O_2_^+^) using a mass spectrometer positioned in front of the surface inside a Feulner cup. The 2 mm aperture of the Feulner cup ensures spatial selectivity, capturing desorbing O_2_ exclusively from either the (111) or (332) facet of the bifaceted crystal. Recombinatively desorbing O_2_ from subsurface sites appears as a sharp, intense peak near 800 K, which increases in concert with AO exposure. In addition to this sharp peak, there is a broad desorption feature up to 1275 K that arises from recombinative desorption of chemisorbed oxygen (O_ad_). This assignment is confirmed by a reference TPD spectrum after molecular O_2_ exposure, which is known to produce only O_ad_ (purple line in Figure 2a, panel a). It lacks the 800 K peak and shows only the broad surface desorption feature. In Figure 2b, the integrated TPD peak areas are plotted as a function of AO exposure. The integrals are referenced to the purple spectrum in Figure 2a, which provides the standard oxygen coverage (*θ_O_*) of 0.5 ML O. The (332) surface shows a higher oxygen uptake than the (111) surface at comparable exposures, indicating enhanced O_sub_ formation on stepped facets. We use the data from Figure 2b as a reference to conduct CO oxidation experiments at controlled and well‐defined oxygen coverages.

**FIGURE 3:**
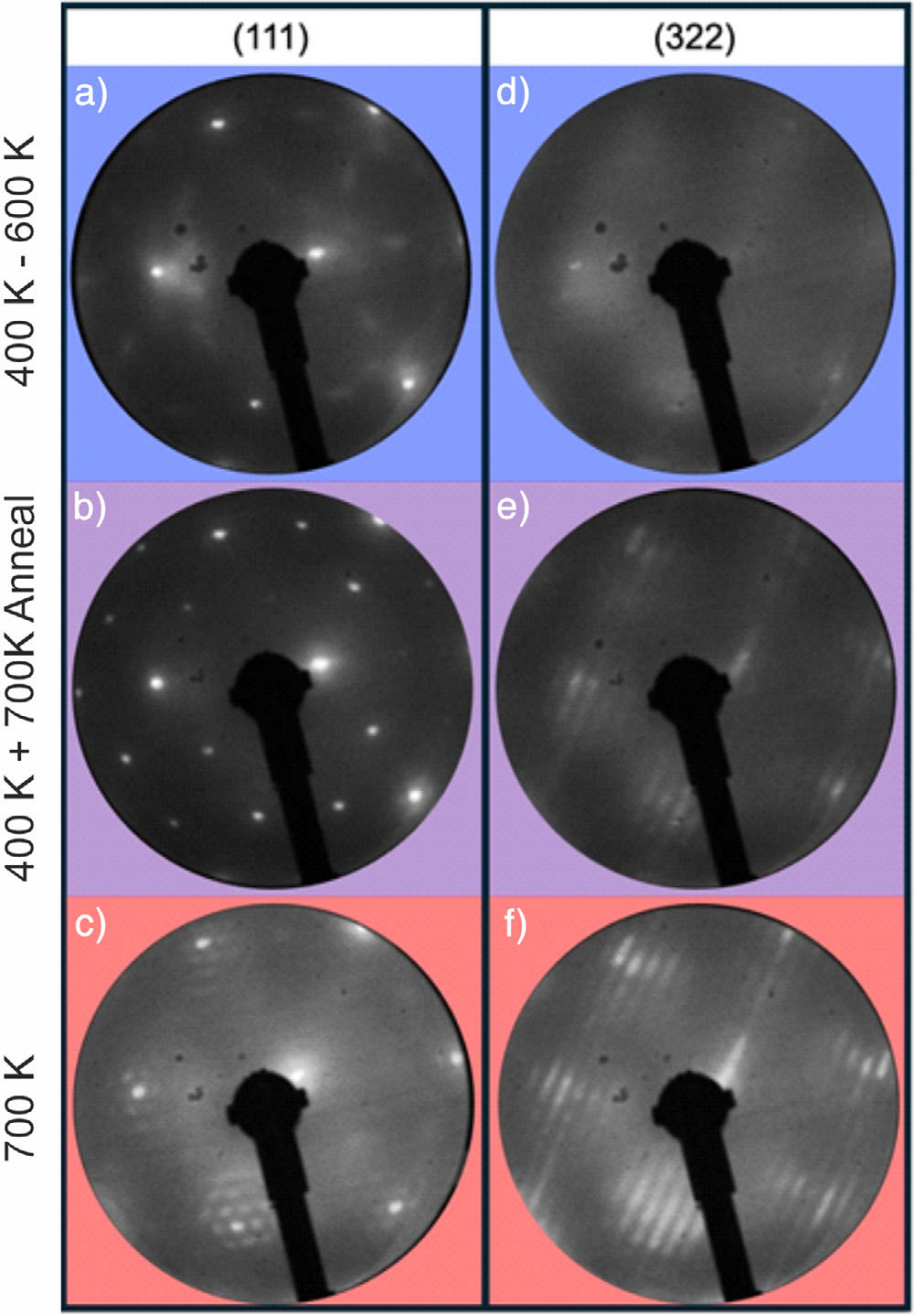
LEED images after AO dosage. The left column is LEED patterns from Rh(111) and the right column from Rh(332). a), d) LEED images after AO dosage between 400–600 K. On Rh(111), the coexistence of the (2√3 × 2√3)*R*30° and (2 × 2)‐3O structure is visible. The steps induce a splitting of the spots on the (332) facet, so that the structure is not clearly resolved. b), e) LEED image after identical dosage as in the top row. However, the surface was heated after the dosing to 700 K. This induces a change to a (2 × 1)‐O pattern on the (111) surface, and fractions of RhO_2_ form on the stepped surface. c), f) LEED images after AO dosage at 700 K. The typical pattern of RhO_2_ is visible for both facets. The electron beam energy at which the LEED was recorded is always 108 eV.

The surface structure of the oxidized surfaces is determined using low‐energy electron diffraction (LEED), as shown in Figure 3. When Rh(111) is oxidized with atomic oxygen between 400–600 K, a coexistence of the (2√3 × 2√3)*R*30° and (2 × 2)‐3O structures is observed, as reported previously [63, 77]. On the stepped Rh(332) facet, spot splitting occurs, making the structure less well‐resolved (Figure 3, top row). Heating the surface to 700 K after dosing at 400 K transforms the coexisting oxygen phases on Rh(111) into a (2 × 1)‐O structure (middle row) [55, 77]. Direct dosing at 700 K results in significantly more subsurface oxygen and the formation of large RhO_2_ domains, producing a characteristic LEED pattern of RhO_2_ on both facets (bottom row) [62, 79]. All experiments in this study were conducted at a dosing temperature of 400 K with subsequent annealing to consistently prevent RhO_2_ formation.

Table 1 summarizes the oxygenated Rh(111) surface obtained under various preparation schemes and temperatures, highlighting the formation of subsurface oxygen (O_sub_), the occurrence of RhO_2_, the associated surface structures, and the maximum attainable oxygen coverage [55, 64, 80]. Although the Rh(332) surface is structurally more complex, its overall behavior regarding O_sub_ formation and RhO_2_ development is similar, as shown in Figure 3. All experiments in this study were conducted at the maximum attainable coverage and under conditions where no RhO_2_ was present by dosing at 400 K with subsequent annealing. The conditions under which the VMI experiments in this study have been performed are indicated by asterisks. Note that heating the surface to 700 K after dosing at 400 K transforms the coexisting oxygen (2√3 × 2√3)*R*30° and (2 × 2)‐3O phases into a (2 × 1)‐O structure.

**TABLE 1:** Maximum attainable oxygen atom coverage (*θ*_O, max_), surface structures, and the formation of rhodium oxide or subsurface oxygen (O_sub_) on Rh(111) after exposure to atomic oxygen (AO) or molecular oxygen (MO) for different oxidants and dosing temperatures (*T*_dos_). The Rh(332) surface exhibits a more complex structure, but its overall behavior regarding subsurface oxygen formation and RhO_2_ development remains similar, as illustrated in Figure 3. The conditions under which the VMI experiments in this study have been performed are indicated by asterisks. All experiments containing subsurface oxygen in this study were conducted at a dosing temperature of 400 K with subsequent annealing to consistently prevent RhO_2_ formation.

Oxidant	*T*_dos_	*θ*_O, max_	Structure	Rhodium oxide	O_sub_
Molecular O_2_ *	400–700 K	0.5 ML	(2 × 1)‐O	None	None
Atomic oxygen	400–600K	0.66^a^–0.75 ML^b^	(2√3 × 2√3)*R*30° and (2 × 2)‐3O	None	Yes
Atomic oxygen	700 K	0.5 ML	(2 × 1)‐O + RhO_2_	Yes	Yes
Atomic oxygen*	400 K, anneal to 600 K	0.66^a^–0.75 ML^b^	(2√3 × 2√3)*R*30° and (2 × 2)‐3O	None	Yes
Atomic oxygen*	400 K, anneal to 700 K	0.5 ML	(2 × 1)‐O	None	Yes
Atomic oxygen*	400 K, anneal to 800 K	0.5 ML	(2 × 1)‐O	None	None

^a^ (2√3 × 2√3)*R*30°.

^b^ (2 × 2)‐3O.

^*^ The conditions under which the VMI experiments in this study have been performed are indicated by asterisks.

### 2.2 Velocity Distributions of Desorbing CO_2_ After CO Oxidation on Oxidized Rh

We investigate the CO oxidation reaction on oxidized Rh surfaces using the general approach illustrated in Figure 1. The Rh crystal is oxidized either via exposure to molecular oxygen from a molecular beam (producing O_ad_) or to atomic oxygen (AO) generated by a hot Ir filament (creating RhO_2_, O_sub_, and O_ad_, depending on the surface temperature during exposure). A second nozzle introduces the CO reactant. At the selected temperatures, CO reacts with oxygen on the surface to form CO_2_, which desorbs immediately. The laser ionization pulse and the CO molecular beam pulse are synchronized in such a way that the desorbing reaction product CO_2_ is ionized and detected by the VMI detector.

For all molecular‐beam experiments, the Rh crystal is prepared with a full monolayer of oxygen, and all measurements are performed at this coverage unless stated otherwise. During the measurements, the coverage remains high because of the low CO concentration in the beam and low reaction probabilities. Over the averaging time for the velocity distributions, the coverage changes only marginally—from 100% to 92% of a monolayer (see Figure S14).

Note that the precise coverage corresponding to a saturated adlayer depends on temperature and the preparation procedure (see Table 1). Unless stated otherwise, all experiments were conducted at 600 K to prevent CO reactant accumulation. For subsurface oxygen preparation, the surface was exposed to atomic oxygen at 400 K and subsequently annealed to 600 K/700 K, ensuring consistent avoidance of RhO_2_ formation.

**FIGURE 4:**
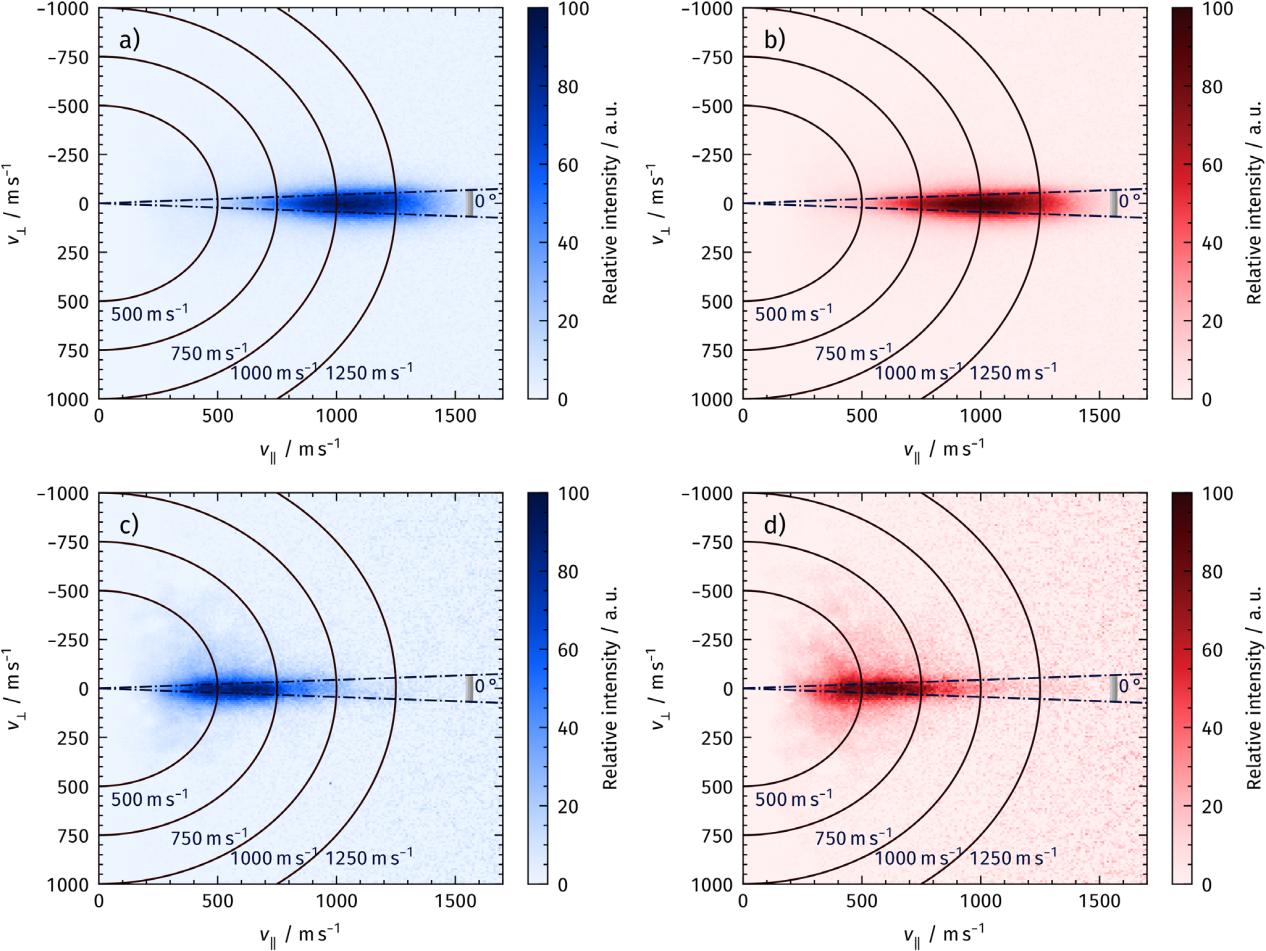
Velocity map images of CO_2_ produced in molecular beam CO oxidation experiments on Rh(111) (blue, panel a) and Rh(332) (red, panel b) at 600 K, shown after converting measured density to flux. The zero‐velocity position is determined from the symmetric background signal on the phosphor screen, which also serves for detector calibration. The velocity components 𝑣_⟂_ and 𝑣_∥_ are defined relative to the surface normal. Velocities are extracted from a 5°‐broad angular slice centered on the surface normal, as indicated in the image. Panels c) and d) show CO_2_ velocity map images of the same reaction, now on Rh surfaces containing both surface‐bound and subsurface oxygen. The velocities are clearly shifted toward lower values.

Figure 4 presents velocity map images of desorbing CO_2_ produced by CO oxidation on Rh(111) and Rh(332), both with and without subsurface oxygen in addition to surface‐bound oxygen. The images show the ion impact positions on the VMI detector, which, after calibration, are directly mapped to velocities relative to the surface normal, as indicated by the axes. A clear shift toward lower velocities is observed in the presence of subsurface oxygen for both surfaces. Rh with subsurface oxygen is prepared as described above and shown in the top row of Figure 3. The surface is dosed at 400 K with AO with subsequent annealing to 600 K (Line 4, Table 1), producing a mixture of (2√3 × 2√3)*R*30° and (2 × 2)‐3O surface phases. This corresponds to *θ*_O_ = 0.65 ML [64].

**FIGURE 5:**
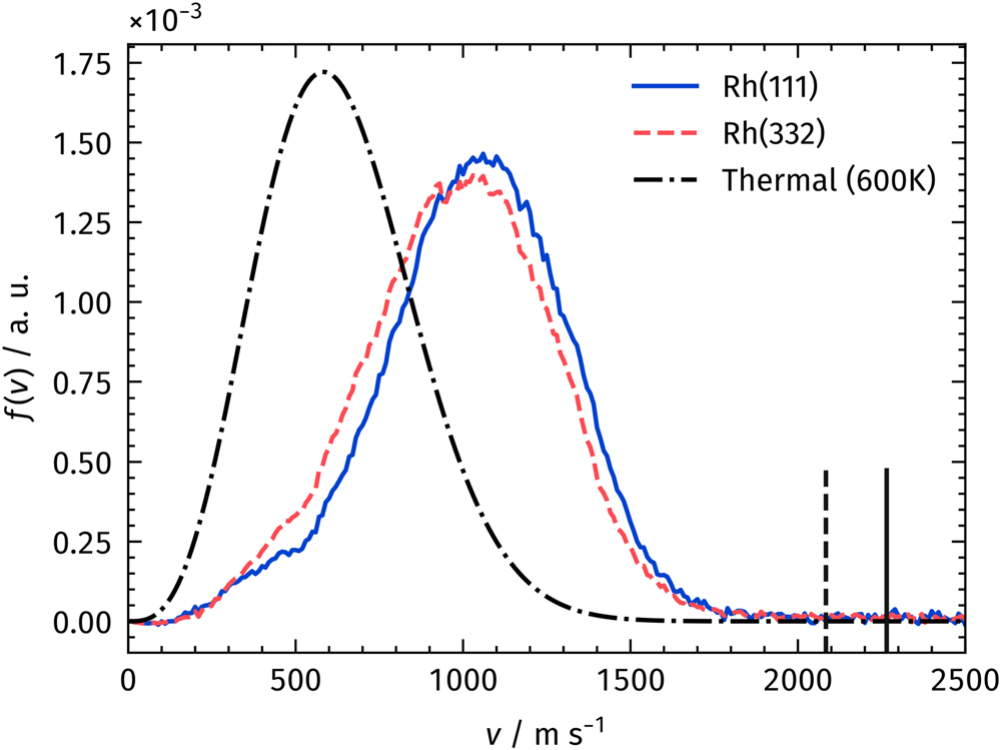
Velocity distributions of desorbing CO_2_ produced by CO oxidation on Rh(111) and Rh(332) at 600 K. Only O_ad_ is present under these conditions. A 600 K thermal distribution is shown for comparison. The data clearly reveal hyperthermal desorption for both surfaces. For reference, the solid (Rh(111)) and dashed (Rh(332)) lines indicate the velocities available from the transition state of the surface reaction, as obtained from the DFT calculations (Figure 9), assuming that all available energy is converted into translational energy along the desorption coordinate.

Velocity distributions were extracted from the VMI images by integrating the signal within the angular segment marked by dashed lines in Figure 4. Figure 5 shows the extracted velocity distributions of desorbing CO_2_ produced by CO oxidation on Rh(111) and Rh(332) when only surface oxygen is present after preparing a (2 × 1)‐O phase with molecular oxygen at 600K (Line 1, Table 1). Compared to the Maxwell‐Boltzmann distribution corresponding to 600 K, the CO_2_ product velocity distributions are clearly shifted toward hyperthermal velocities.

The reaction enthalpy of CO oxidation is 2.93 eV, which would correspond to a desorption velocity of approximately 3580 m/s if all the released energy were converted into the translational desorption motion of the CO_2_ product. This velocity is far higher than what we observe experimentally. A more realistic comparison is therefore to use the available translational energy at the transition state of the surface reaction, as obtained from the DFT calculations and shown in Figure 9, following the approach used previously [81]. The corresponding CO_2_ velocities are indicated as lines in Figure 5. If the full available translational energy from the transition state were transferred solely into the desorption coordinate, we would expect a narrow distribution centered at that velocity. Instead, the measured distributions are broad and shifted to lower velocities. This demonstrates that a significant portion of the reaction energy is dissipated into other degrees of freedom, including vibrational and rotational excitation of the product as well as energy transfer to lattice phonons.

**FIGURE 6:**
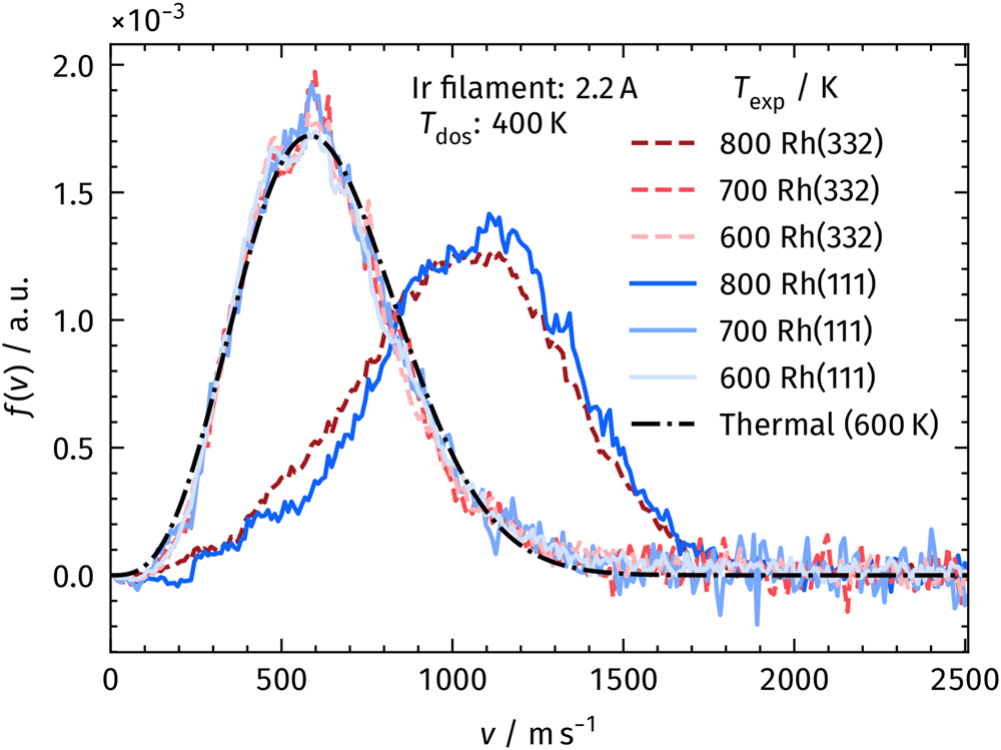
Velocity distributions of desorbing CO_2_ from CO oxidation on Rh(111) (solid, blue) and Rh(332) (dashed, red) in the presence and absence of O_sub_. The oxidized surfaces were prepared by exposing the samples to AO at 400 K for several minutes, yielding a total oxygen coverage of approximately 2 ML. Here, the term “coverage” includes both surface oxygen and subsurface oxygen formed during AO exposure. After oxidation, the surfaces were ramped to 600 K, 700 K, or 800 K, resulting in oxygen overlayers with (2√3 × 2√3)*R*30° and (2 × 2)‐3O (600 K) and (2 × 1)‐O (700 K, 800 K) structures while preventing the formation of RhO_2_ (Table 1). At 600 and 700 K, the velocity distributions are thermal, but a pronounced shift to hyperthermal velocities occurs at 800 K. This change is attributed to the desorption of O_sub_ near 800 K (see Figure 2), resulting in CO oxidation occurring exclusively on surface‐bound oxygen, which produces hyperthermal CO_2_ desorption. See Figure 5.

The dramatic change of the product CO_2_ velocity distributions in the presence of O_sub_ is shown in Figure 6. The corresponding data in Figure 4c,d show that the CO_2_ desorption velocities after CO oxidation on both Rh(111) and Rh(332) are notably slower in the presence of O_sub_. The velocity distributions in Figure 6 clearly demonstrate the shift from thermal distributions, which correspond to the surface temperature when O_sub_ is present, to the hyperthermal distributions once O_sub_ is depleted, and only O_ad_ is available to oxidize the incident CO. The Rh surface is first oxidized with AO at 400 K, forming both O_sub_ and O_ad_. The resultant surface was then warmed to either 600 K, 700 K, or 800 K, and then exposed to the CO beam, and the product CO_2_ velocity distribution is collected.

O_sub_ is present at both 600 K and 700 K, but the surface phase shifts from the high‐density phase (2√3 × 2√3)*R*30° / (2 × 2)‐3O to the (2 × 1)‐O at 700 K. Even though the surface phase change, we do not observe a change in the thermal velocity distribution. At 800 K, O_sub_ is no longer present, and only the (2 × 1)‐O phase remains (see Table 1). It is evident that the CO_2_ product is not significantly altered going from 600 K to 700 K, but the distribution is markedly different at 800 K. When O_sub_ is present, the velocity distributions are thermal, and when it has been depleted, the distribution returns to being hyperthermal. It is altogether clear that O_sub_ changes the reaction mechanism, and it is interesting to note that both facets display the same behavior.

**FIGURE 7:**
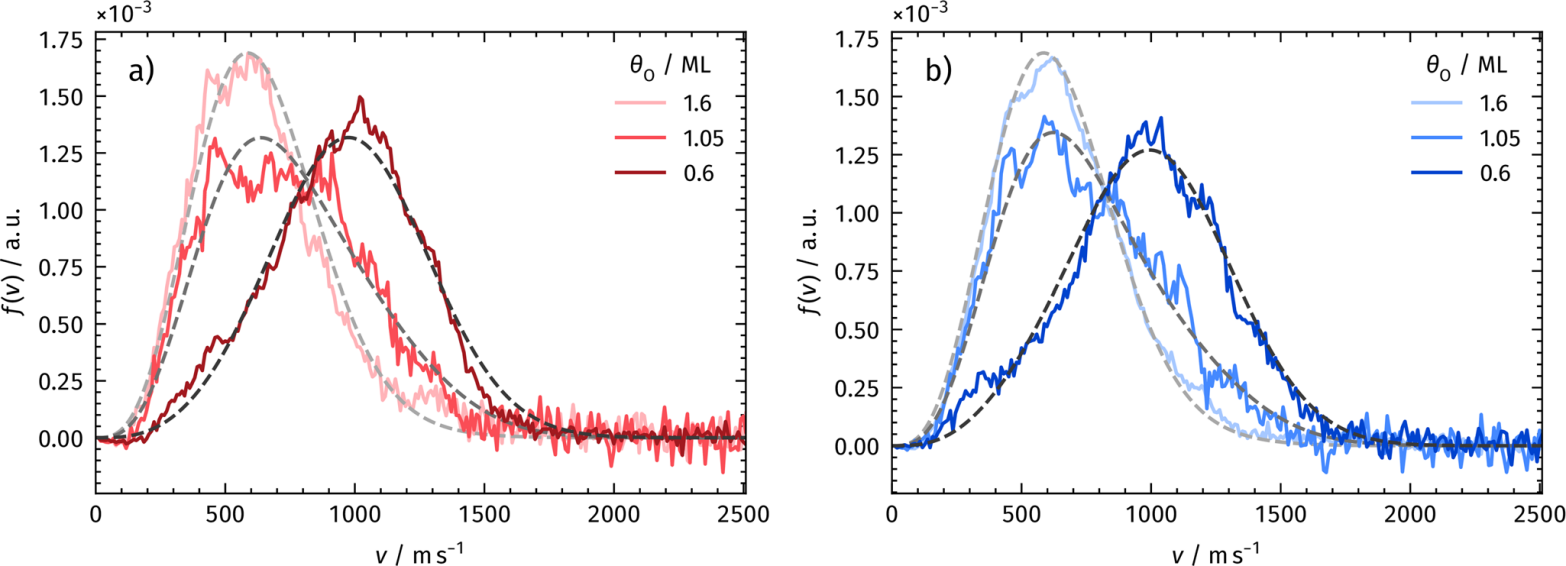
Velocity distributions of desorbing CO_2_ reaction product at three oxygen coverages on Rh(111) a) and Rh(332) b). Here, “coverage” includes both surface oxygen and subsurface oxygen formed during atomic oxygen (AO) exposure. Both surfaces show similar behavior: at low coverage, the distributions are hyperthermal and progressively evolve toward a thermal distribution as the oxygen coverage increases. The oxidized surfaces were prepared by exposing the samples to AO at 400 K for defined times, with the resulting coverages determined by TPD experiments (Figure 2). After oxidation, the surfaces were ramped to 600 K, producing oxygen adlayers with (2√3 × 2√3)*R*30° and (2 × 2)‐3O structures while avoiding RhO_2_ formation (Table 1). At this temperature, CO oxidation experiments have been performed.

In a separate experiment, we examine how the velocity distribution of desorbing CO_2_ reaction products depends on oxygen coverage. TPD measurements quantified the coverage after defined exposure times to AO (Figure 2). Note that the term “coverage” also includes O_sub_ formed during AO exposure. For the experiments, the precisely quantified oxidized surfaces were exposed to a pulsed molecular beam of CO, and the resulting CO_2_ velocity distributions were recorded. As shown in Figure 7, the distributions evolve from hyperthermal at low *θ*_O_ to thermal at higher *θ*_O_, with intermediate *θ*_O_ displaying a mixed character. We observe this behavior for both the (111) and the (332) surface.

**FIGURE 8:**
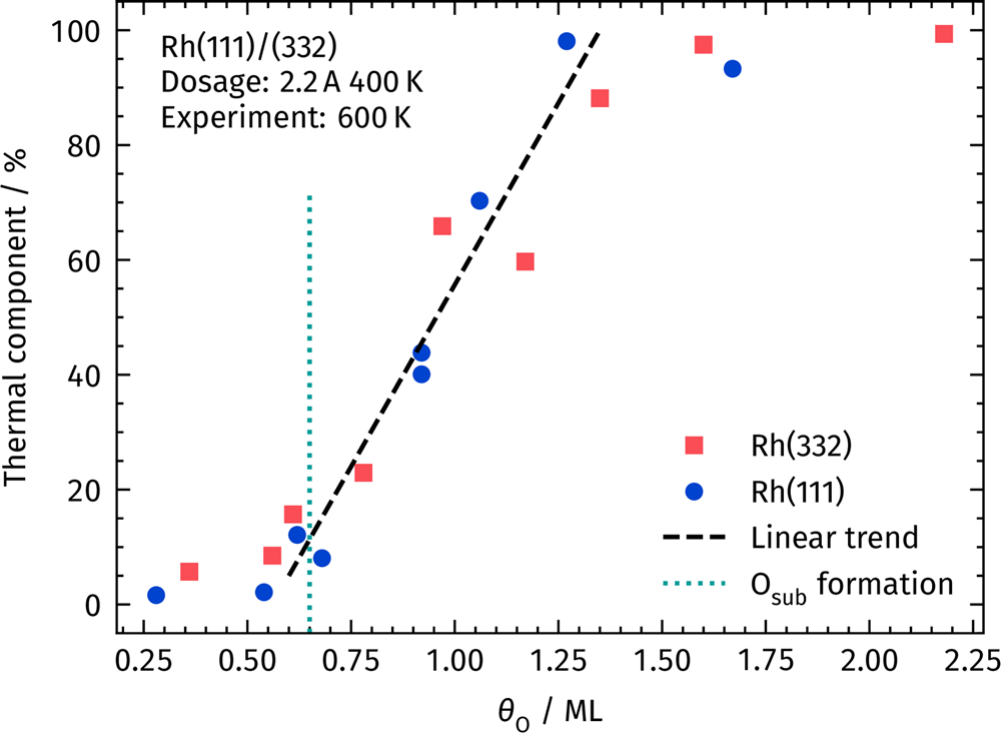
Fraction of thermal CO_2_ in the velocity distribution *f*(*v*) as function of 𝜃_O_ after AO dosage at 400 K. When O_sub_ uptake begins at *θ*_O_ = 0.65 ML, a linear increase of the fraction can be seen, until a thermal distribution is resembled by the data. The dashed black line is drawn to guide the eyes. The dotted green line denotes *θ*_O_ = 0.65 ML, with any further oxygen uptake corresponding to subsurface oxygen formation.

In Figure 7, we fit the experimental data with a sum of two components: a thermal and a hyperthermal distribution. The thermal component is modeled using a flux‐weighted Maxwell‐Boltzmann distribution, while the hyperthermal component is fit by a flowing flux‐weighted Maxwell‐Boltzmann distribution introducing a velocity shift *v*_0_ [44, 56]:

(1) $$f\left(v,T\right)\propto \alpha \frac{v^{3}}{T\sqrt{T}}e^{-\frac{Mv^{2}}{2RT}}+\left(1-\alpha \right)\frac{v^{3}}{T\sqrt{T}}e^{-\frac{M{\left(v-v_{0}\right)}^{2}}{2RT}}$$

here *v* is the velocity, *T* is the temperature, *M* is the carbon dioxide mass, and *R* is the molar gas constant. The parameter α specifies the fractional contribution of the thermal component to the data. Figure 8 shows the fractional contribution of thermal CO_2_ in the velocity distribution as a function of oxygen coverage. Once saturation of the mixed (2√3 × 2√3)‐O and (2 × 2)‐3O surface phase on Rh(111) is reached (*θ*_O_ = 0.65 ML) [64], thermal CO_2_ appears in the distribution and increases linearly until, at around *θ*_O_ = 1.25 ML, only thermal CO_2_ remains.

We conclude from the experimental data that subsurface oxygen profoundly alters the reaction pathway, producing markedly different dynamics. To elucidate the underlying reasons, we employ density functional theory (DFT), to model the system.

### 2.3 DFT Calculations on Reaction Pathways of CO Oxidation on Oxidized Rh

In a pioneering study, Neugebohren et al. investigated CO oxidation on Pt surfaces, applying velocity‐resolved kinetics measurements based on ion imaging techniques for the first time [37]. They found that CO_2_ desorbs hyperthermally from terraces, whereas desorption from step sites occurs thermally. In a follow‐up work, DFT‐based *ab initio* molecular dynamics simulations were used to explain this behavior [19]. The simulations revealed that on stepped Pt surfaces, CO_2_ desorption follows the formation of an intermediate chemisorbed state in the post‐transition state reaction coordinate, created by electron transfer from the surface to an antibonding CO_2_ orbital. As a consequence, bent CO_2_ traps at the surface. This trapping in the chemisorption well leads to thermalized velocity distributions of desorbing CO_2_. On Pt(111) terraces, however, the chemisorption well is insufficiently stabilized, so CO_2_ desorbs directly without intermediate trapping after the transition state, producing high velocities as reaction energy is channeled into the desorption coordinate.

Building on the Pt surface study, we employ the same DFT methodology to investigate the reaction pathways of forming CO_2_ following CO oxidation on Rh(111) and Rh(332), with and without subsurface oxygen. Computational details of the DFT setup are given in the Supporting Information.

**FIGURE 9:**
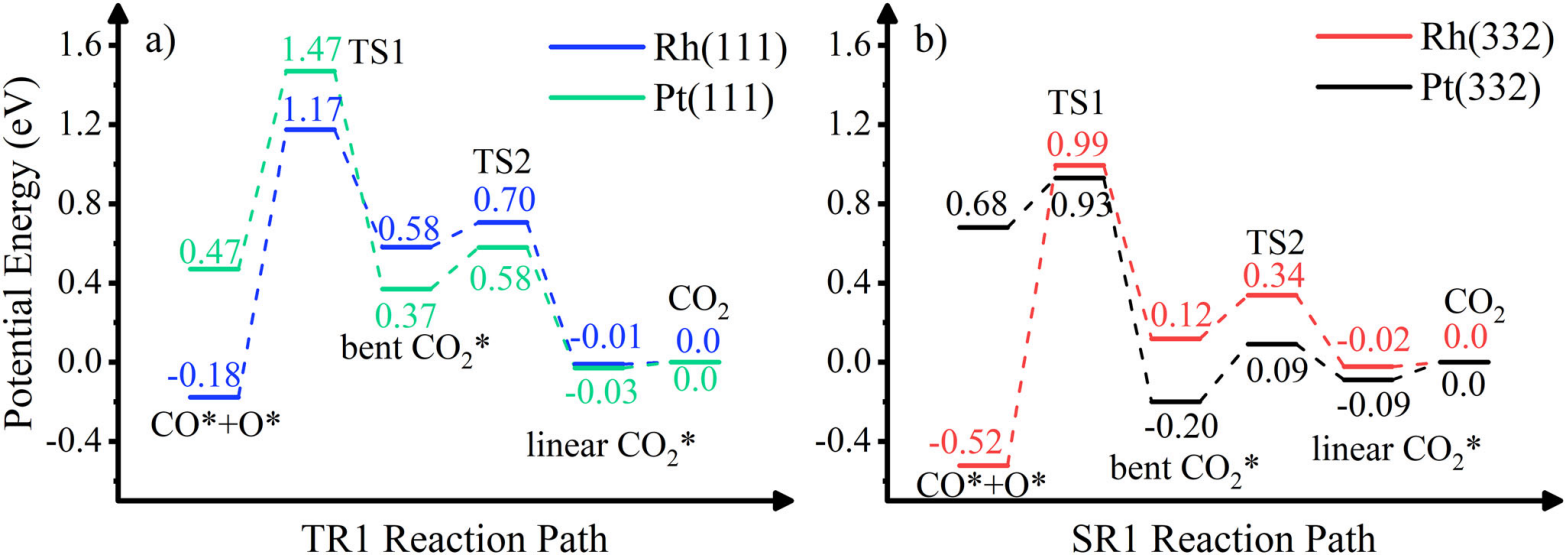
Energetics of CO oxidation pathways (TR1 and SR1) on Pt and Rh surfaces, referenced to non‐interacting CO_2_. The TR1 pathway corresponds to reactions occurring at terrace sites, while the SR1 pathway represents reactions at step sites.

Following the study of CO oxidation on Pt surfaces, TR1 and SRX (X = 1–4) denote the terrace reaction and step reactions, respectively. Figure 9a compares the energetics of two CO oxidation reaction paths (TR1 and SR1) on Rh and Pt, with energies referenced to the free CO_2_ and surface. In TR1, the reaction pathway is bottlenecked by an initial transition state (TS1) corresponding to CO_2_ formation at the surface. Beyond this point, a chemisorption well appears, associated with a bent CO_2_ molecule formed via electron transfer from the surface to an antibonding CO_2_ orbital. This intermediate state is more stabilized on Pt than on Rh, yet remains shallow on both surfaces relative to the second transition state (TS2), leading to desorption. It is also not energetically favorable with respect to the final energetics of CO_2_ + metal, so that no trapping occurs and final velocities of desorbing CO_2_ are hyperthermal. Notably, the overall reaction is slightly endothermic on Rh(111) but exothermic on Pt(111).

In contrast, stepped surfaces exhibit markedly different energetics. Figure 9b shows the most favorable CO oxidation pathway on a stepped Pt(332) surface, referred to as SR1 in Ref. [19]. Compared to the terrace reaction, the barrier to TS1 is significantly lower. Beyond this point, the bent CO_2_ product is stabilized at the step site by ∼0.2 eV relative to desorbed CO_2_. This stabilization traps CO_2_ in the chemisorption well long enough for thermal accommodation. Desorption from this state via TS2 then proceeds thermally, with a broad angular distribution, as confirmed by *ab initio* molecular dynamics simulations [19].

We also analyze CO oxidation pathways on Rh(332), for which a similar SR1 pathway is presented in Figure 9b, while additional pathways are shown in Figures S2–S6. It is clear that CO oxidation still needs to overcome a high barrier over TS1, followed by a shallow chemisorption well relative to TS2. Unlike Pt, no pathway on Rh exhibits a bent CO_2_ chemisorption well stabilized relative to desorbed CO_2_. Consequently, the post‐transition state dynamics are expected to differ: CO_2_ is not trapped for thermal accommodation but instead desorbs directly and rapidly from the first transition state. This is consistent with our experimental observation of exclusively hyperthermal velocity distributions for both the (332) and (111) surfaces.

**FIGURE 10:**
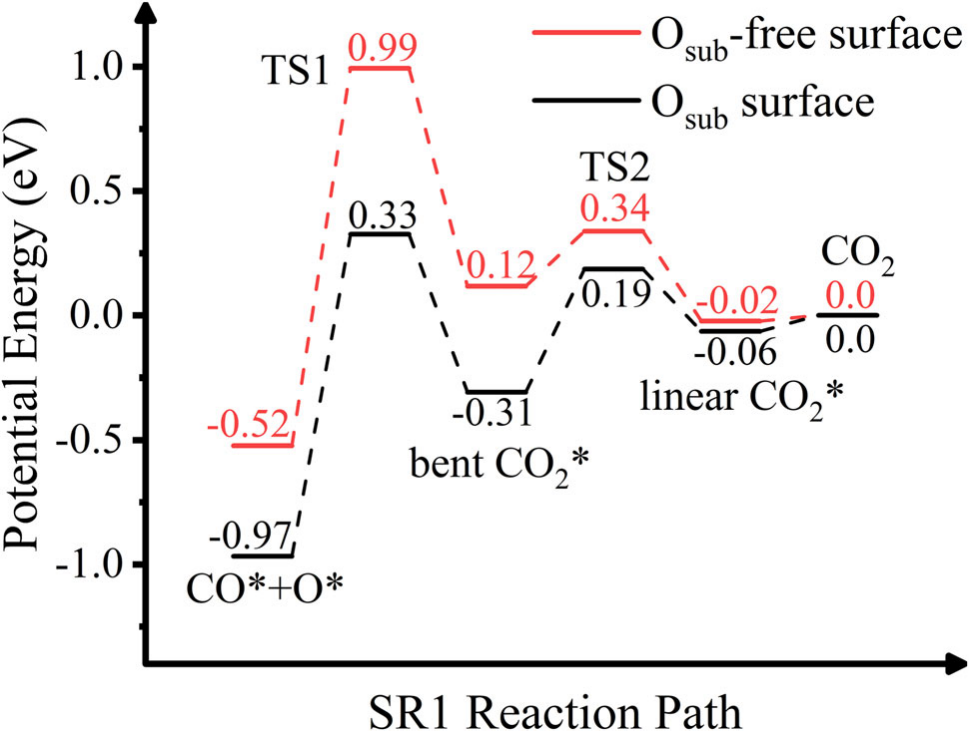
Energetics of an SR1 CO oxidation pathway on a Rh(332) surface with subsurface oxygen and on a surface without subsurface oxygen (O_sub_‐free). CO reacts with surface oxygen, while subsurface oxygen acts as a spectator, modifying the potential energy surface. Compared to Rh without subsurface oxygen, the adsorbed reactants, as well as the post‐transition state adsorbed bent CO_2_ product are stabilized relative to gas‐phase CO_2_ and metal. This results in a much deeper chemisorption well relative to TS2 (∼0.50 eV), enabling sufficient trapping of the bent CO_2_ adsorbate for thermalization.

The presence of O_sub_ significantly alters the reaction energetics. Figure 10 shows the energetics of SR1 CO oxidation pathway on Rh(332), where CO reacts with surface oxygen while O_sub_ acts as a spectator, modifying the PES. Compared to Rh surfaces without O_sub_, both the coadsorbed reactants and the bent CO_2_ are significantly stabilized relative to desorbed CO_2_. This results in a much deeper chemisorption well relative to TS2 (∼0.50 eV), enabling sufficient trapping of the bent CO_2_ adsorbate for thermalization. These findings align with experimental observations, where O_sub_ induces thermal velocity distributions of the desorbing product. In the experiments, the presence of O_sub_ on both Rh(111) and Rh(332) surfaces exhibits similar thermal distributions of the CO_2_ product. Indeed, Rh(111) has a more compact atomic arrangement and a lower structural flexibility than Rh(332). O_sub_ is likely even more difficult to directly react with the CO adsorbate on Rh(111) and is thus expected to influence the CO oxidation pathway in a similar way as on Rh(332).

Considering a possible trajectory from the first transition state to the desorbing reaction product, it is evident that if all of the energy released in overcoming TS1 were converted into CO_2_ translation along the surface normal, the bent‐CO_2_ surface intermediate would be bypassed. We therefore propose that part of the available energy must be redistributed into other degrees of freedom, most likely internal vibrational excitation. In this scenario, even a modest deepening of the bent‐CO_2_ potential well would be sufficient to accommodate the excess translational energy. Similar behavior has been observed experimentally for CO oxidation on platinum surfaces [82] and in theoretical AIMD studies for the same system, where excitation of all three CO_2_ normal modes is observed [19].

In conclusion, O_sub_ exerts a strong indirect influence on the reactivity of Rh surfaces by altering the potential energy surface. Although it does not react directly with CO, the additional oxygen atoms modify the surface charge distribution, enhancing electron transfer from the surface to the adsorbates. Bader charge analysis [83, 84] reveals that, relative to the clean Rh surface, in the SR1 reaction pathway with O_sub_, an additional 0.08 e^−^ and 0.12 e^−^ are transferred from the surface to the CO* + O* reactants and the bent CO_2_ species, respectively. This enhanced charge transfer stabilizes both the reactants and the products on the surface, thus increasing their residence time and allowing for energy equilibration between the nascent products and the surface.

This may influence catalytic performance under real‐world conditions with implications for typical reaction pathways. When considering the hyperthermal, direct‐desorption case, a large fraction of reaction energy is carried away from the surface immediately and redistributed into internal degrees of freedom of the product. In contrast, with subsurface oxygen present, the CO_2_ molecule becomes transiently stabilized in a shallow adsorption well corresponding to a bent CO_2_ geometry. During its residence in this intermediate state, the molecule dissipates energy into the surface, potentially causing local heating and dynamically influencing the surface structure. Moreover, the prolonged residence time at the surface may affect selectivity and reduce turnover frequencies under steady‐state catalytic conditions.

## 3 Conclusion

A combination of experimental techniques and DFT calculations reveal the profound impact of O_sub_ on the heterogeneously catalyzed oxidation of CO on Rh surfaces. The velocity distributions of the CO_2_ products were obtained using molecular beam surface scattering with ion imaging of product CO_2_ desorbing from oxidized Rh surfaces under ultra‐high vacuum conditions, in either the presence or absence of O_sub_. In contrast to CO oxidation on Pt, on both stepped and planar rhodium surfaces, CO_2_ does not thermalize with the surface, indicating direct, rapid desorption and partitioning of the reaction energy into the gas‐phase. Alternatively, when O_sub_ was present, the mechanism is fundamentally altered and the CO_2_ thermalizes with the surface prior to desorption. These results were explained by DFT calculations that show that O_sub_ promotes the formation of a post‐transition state bent CO_2_ species via electron transfer from the surface to an antibonding CO_2_ orbital. Although the initial energy released upon overcoming the first transition state is high, redistribution of energy from the desorption coordinate to internal degrees of freedom enables eventual trapping in a shallow desorption well as bent CO_2_. This stabilizing chemisorption well provides sufficient residence time for thermal accommodation prior to desorption. Altogether, these findings demonstrate the profound impact spectator species play in heterogeneously catalyzed reactions and the need to incorporate them into robust, predictive models of surface catalysis.

## Conflicts of Interest

The authors declare no conflicts of interest.

## Supporting information




**Supporting File 1**: The authors have cited additional references within the Supporting Information [44, 56, 1–10].

## Data Availability

The data that support the findings of this study are available from the corresponding author upon reasonable request.
