# Supplementary material for: Controlling Heterogeneous Catalysis With Subsurface Oxygen
Source: Angew Chem Int Ed Engl. 2026 Feb 17;65(19):e24699. doi: 10.1002/anie.202524699 (PMC13134625; doi:10.1002/anie.202524699)
Supplement: Supplementary file 1 — Supporting File 1: The authors have cited additional references within the Supporting Information [44, 56, 1–10]. [file ANIE-65-e24699-s001.docx]

**Supporting Information**

**S-I. COMPUTATIONAL DETAILS**

All periodic density functional theory (DFT) calculations were performed with the Vienna Ab Initio Simulation Package (VASP).^[91, 92]^ The electron exchange-correlation effects were treated within the generalized gradient approximation (GGA), using the Perdew–Burke–Ernzerhof (PBE)^[90]^ functional. The flat surface of Rh(111) was modeled by a four-layer slab, which has a 3 × 3 unit cell with the top three layers relaxed. The stepped surface of Rh(332) has large (111) terraces with steps spaced by six atomic rows. Here, we used a 1 × 4 supercell surface with four atomic layers, three of which were relaxed. The structures of the Rh(111) and Rh(332) surfaces are shown in Figure S1. The valence electrons were expanded with plane waves with a cutoff energy of 400 eV, while the projector augmented wave (PAW)^[88, 89]^ method was used for the core electrons. The Brillouin zone integration was performed using the k-point grids of 4 × 4 × 1 and 3 × 2 × 1 for the flat and stepped surfaces, respectively. A Fermi-Dirac smearing with a width of 0.1 eV was employed to help convergence. The geometries were optimized using a conjugate-gradient method and the saddle points were determined using the climbing image nudged elastic band (CI-NEB)^[87]^ method and dimer^[86]^ method with force convergence criterium of 0.03 eV·Å^−1^. Bader charge^[83, 84]^ analysis was performed to identify atomic charges.


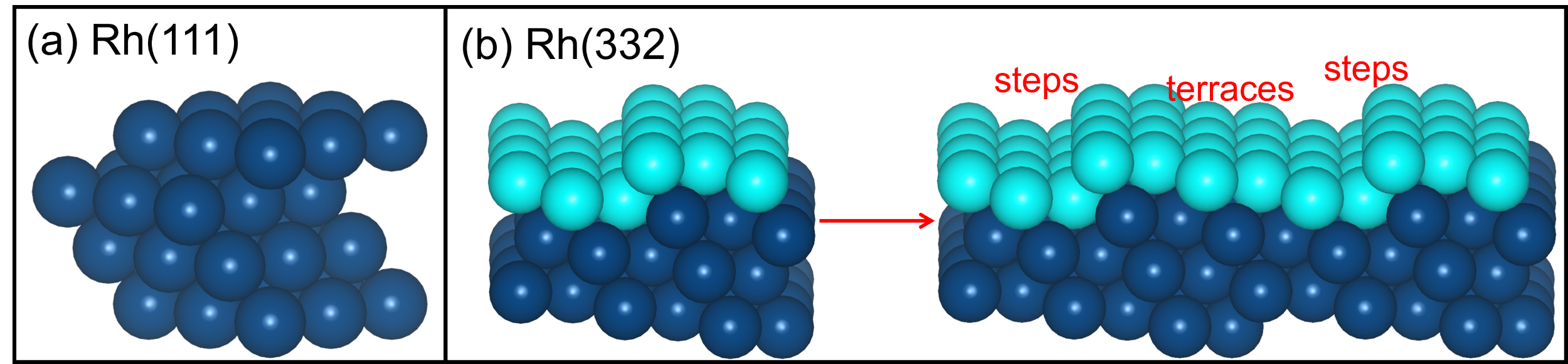


Figure S1. Structural models of the Rh(111) and Rh(332) surfaces used in this study. The periodically expanded Rh(332) model highlights the positions of terraces and repeating step edges. Light blue spheres denote the topmost Rh atoms of the Rh(332) surface, while dark blue spheres represent bulk Rh atoms.

**S-II. ADDITIONAL RESULTS**

**S-IIa. Clean Surface**

Follow the oxidation reaction pathway of CO on Pt to obtain the oxidation reaction pathway of CO on Rh.^[19]^ Figure S2-6 shows the energetics and geometries information for all reaction pathways. More detailed transition state geometries information is shown in Table S1. Note no pathway on Rh exhibits a bent CO_2_ chemisorption well stabilized relative to desorbed CO_2_. Consequently, in post–transition state dynamics, CO_2_ is not trapped for thermal accommodation but instead desorbs directly and rapidly from the first transition state. This is consistent with experimental observation of exclusively hyperthermal velocity distributions for both the (332) and (111) surfaces.


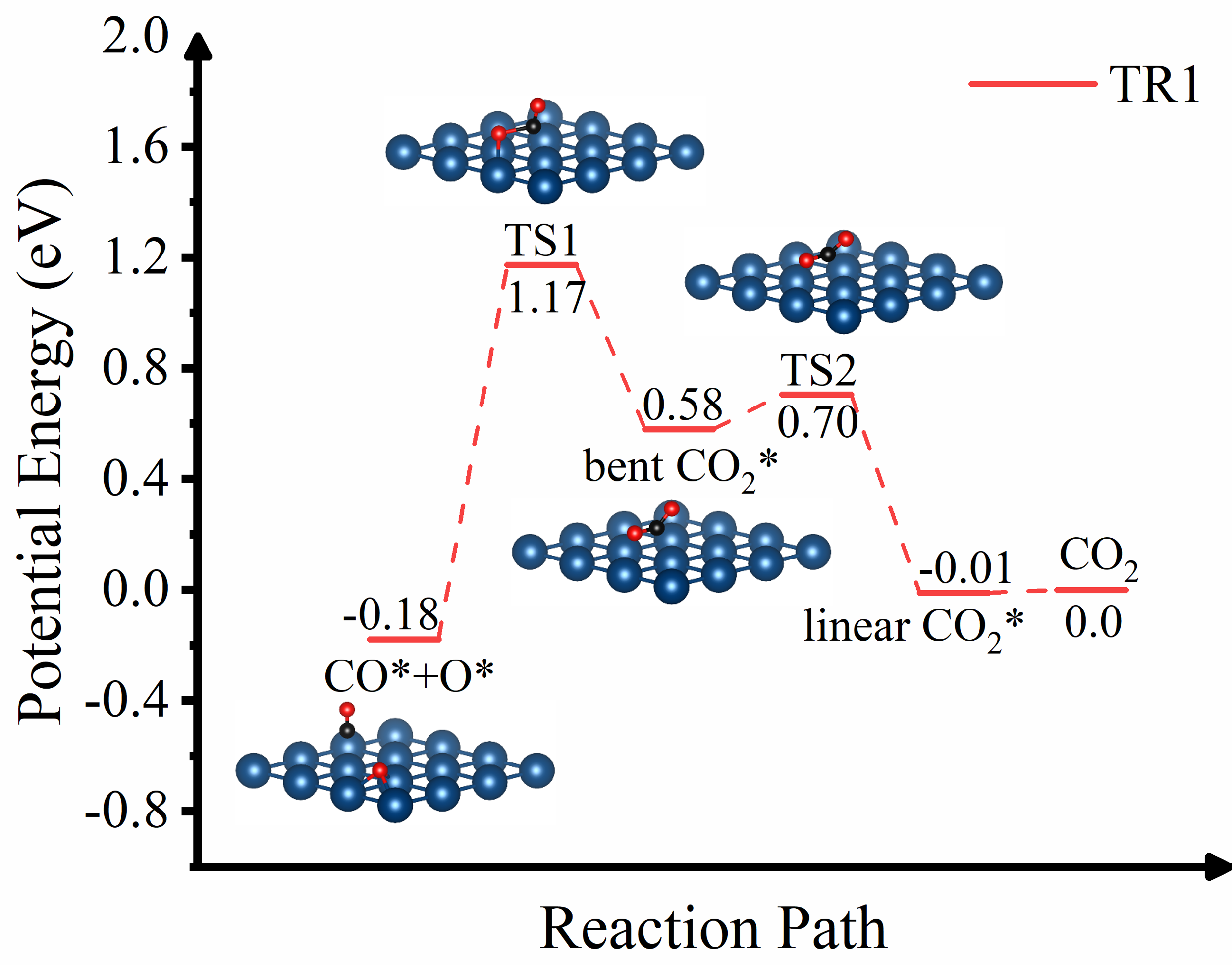


Figure S2. Energetics and geometries of the TR1 Path for CO oxidation on Rh(111).


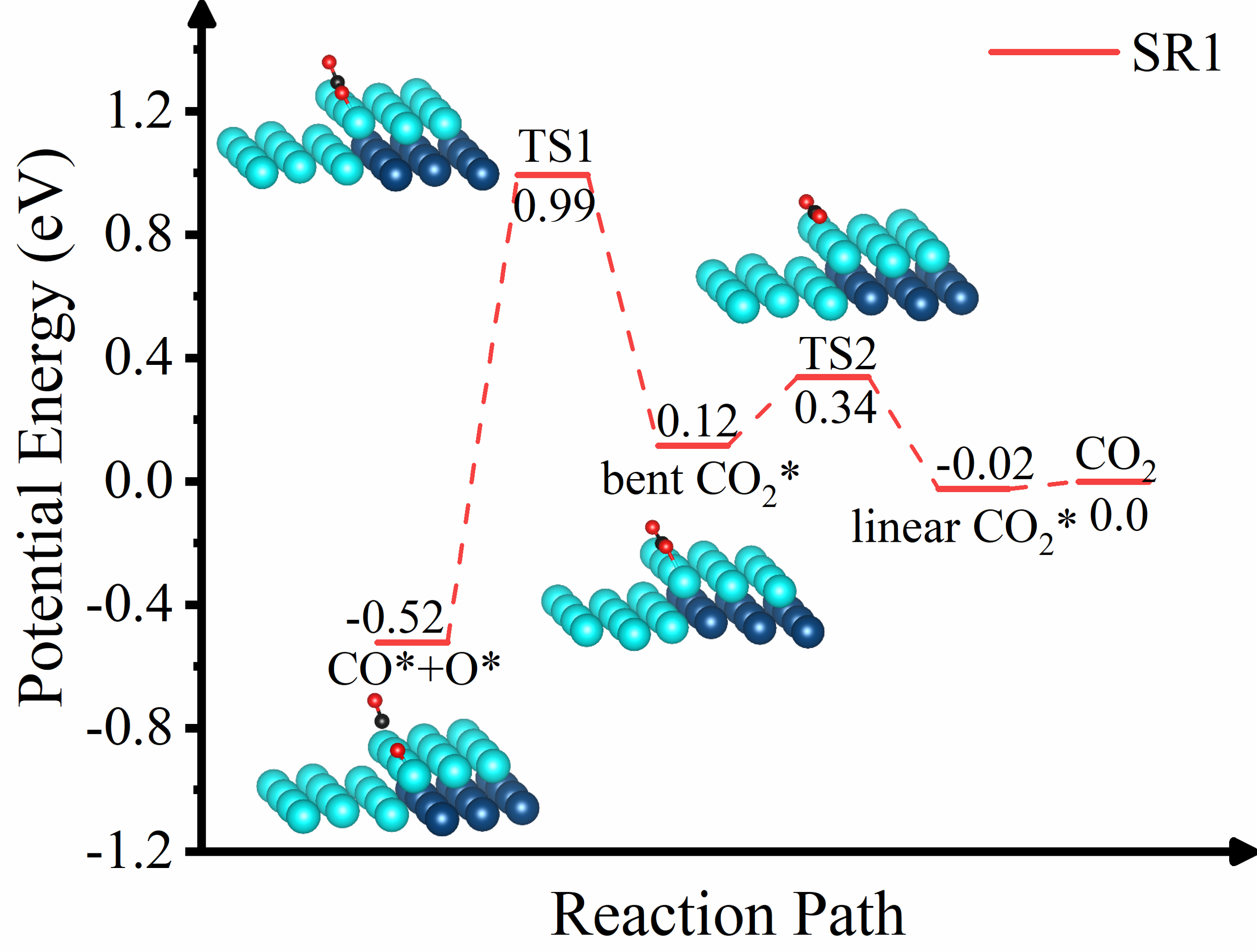


Figure S3. Energetics and geometries of the SR1 Path for CO oxidation on Rh(332).


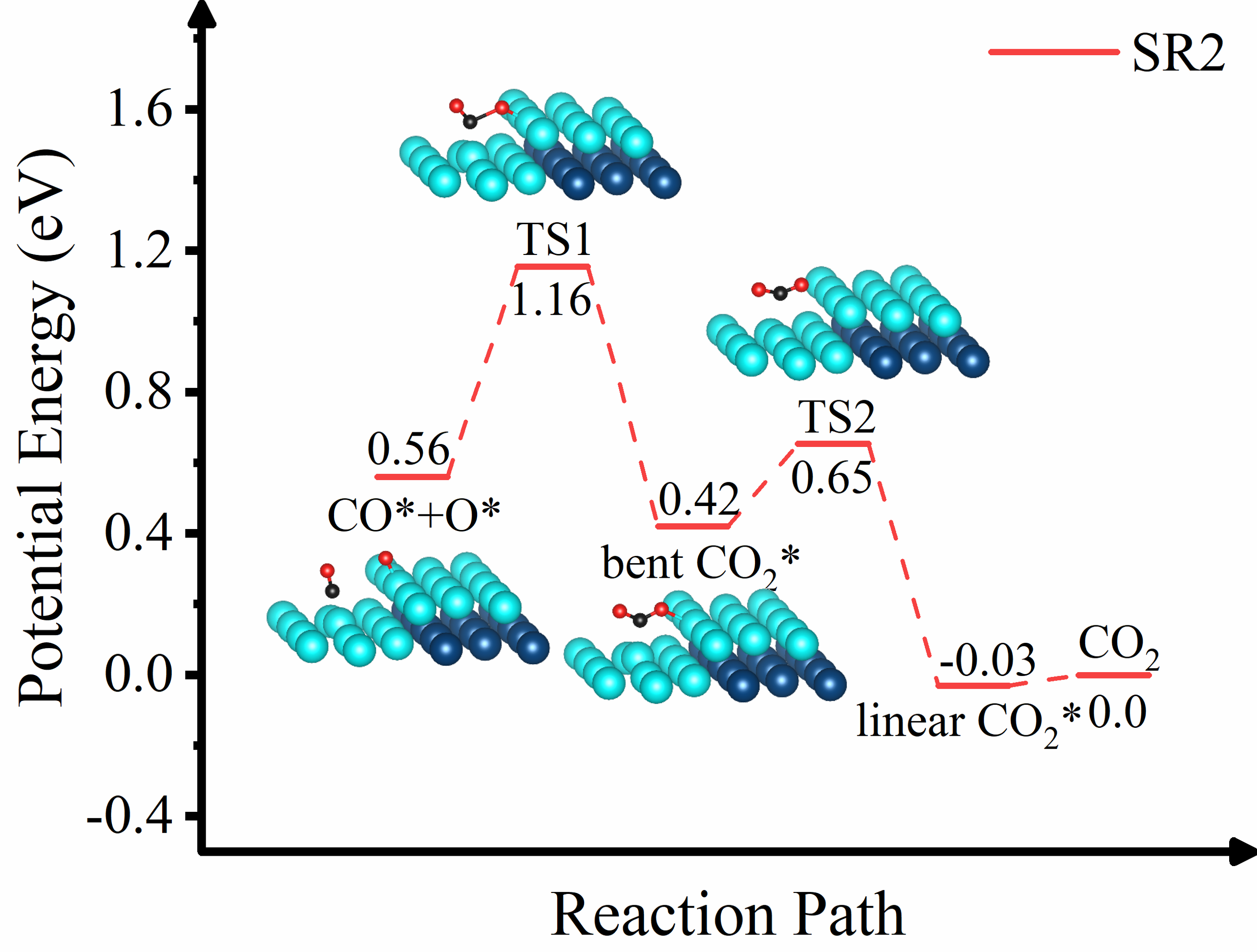


Figure S4. Energetics and geometries of the SR2 Path for CO oxidation on Rh(332).


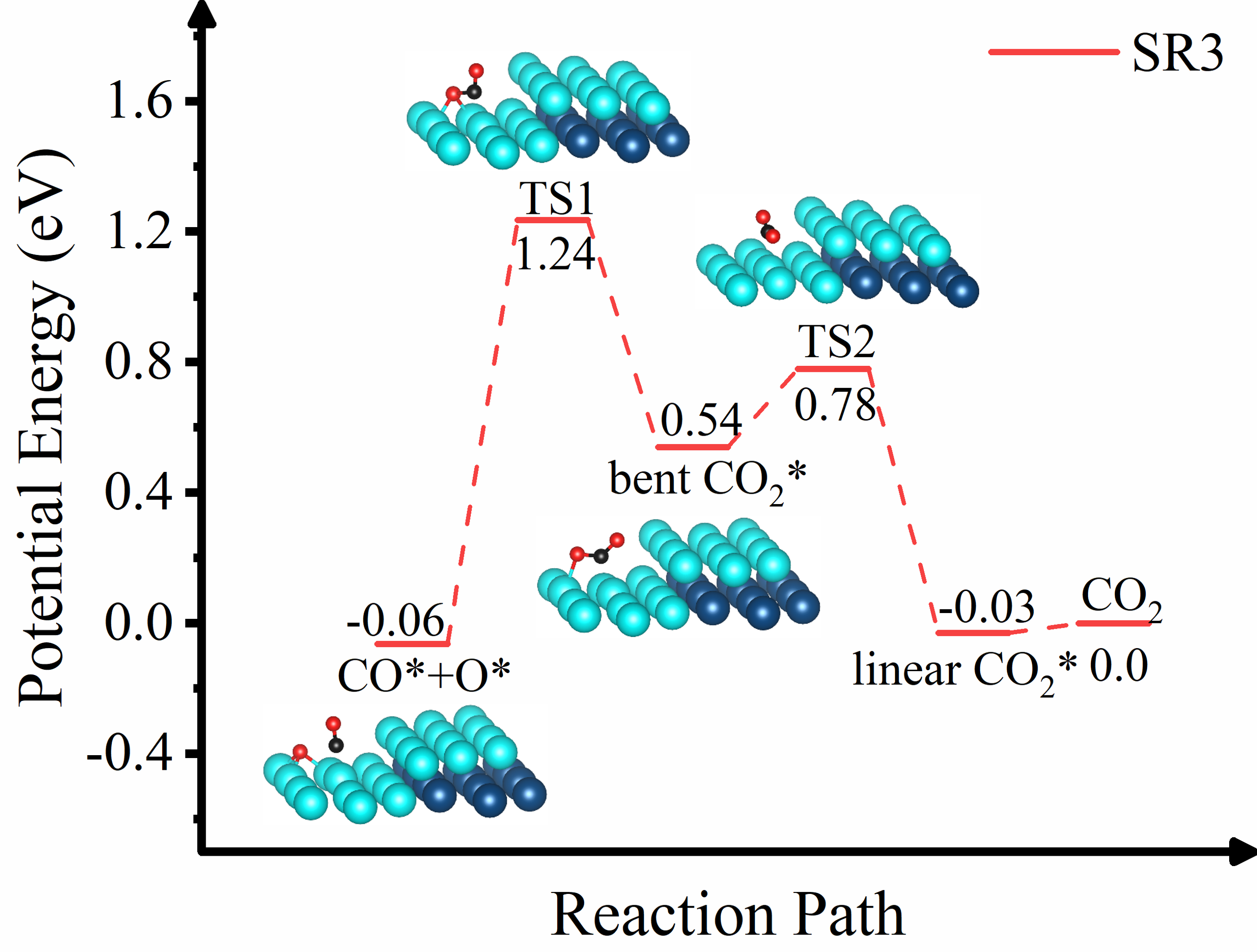


Figure S5. Energetics and geometries of the SR3 Path for CO oxidation on Rh(332).


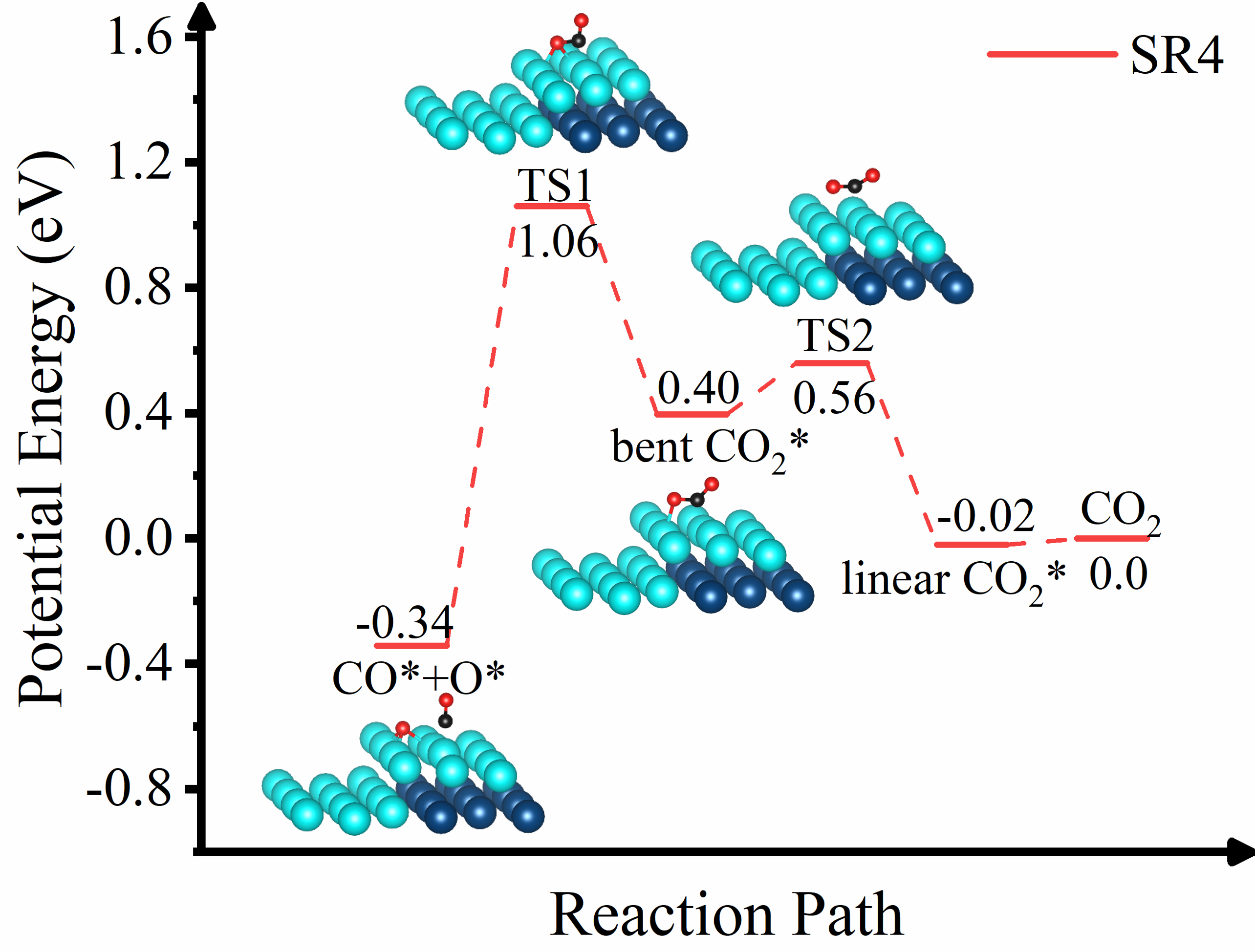


Figure S6. Energetics and geometries of the SR4 Path for CO oxidation on Rh(332).

| Transition state | Reaction Path | *d*_C−Rh_ (Å) | *d*_C−O1_ (Å) | *d*_C−O2_ (Å) | *θ*_OCO_ (deg) |
| --- | --- | --- | --- | --- | --- |
| TS1 | TR1 | 1.96 | 1.77 | 1.18 | 114.07 |
|  | SR1 | 1.96 | 1.84 | 1.18 | 111.90 |
|  | SR2 | 1.95 | 1.89 | 1.18 | 106.95 |
|  | SR3 | 1.96 | 1.81 | 1.18 | 113.20 |
|  | SR4 | 1.96 | 1.78 | 1.18 | 113.74 |
| TS2 | TR1 | 2.48 | 1.22 | 1.20 | 150.25 |
|  | SR1 | 2.56 | 1.21 | 1.20 | 157.26 |
|  | SR2 | 2.59 | 1.22 | 1.20 | 148.14 |
|  | SR3 | 2.43 | 1.22 | 1.20 | 148.32 |
|  | SR4 | 2.55 | 1.22 | 1.19 | 151.59 |

Table S1. Geometric parameters for TS1 and TS2 in different reaction paths in Figure S2-6. *d*_C−Rh_, *d*_C−O1_, and *d*_C−O2_ are the distances of the C−Rh, C−O1, and C−O2 bonds, respectively. *θ*_OCO_ is the OCO bond angle.

**S-IIb. O_sub_ surface**

Note that identifying stable surface structures in the presence of subsurface oxygen (O_sub_) is nontrivial. We therefore performed extensive and systematic tests in which O_sub_ atoms were introduced in varying amounts and initial positions between the stepped layer and the second layer of a clean Rh(332) surface. The number of O_sub_ atoms ranged from a single atom to three atomic rows. No configurations were manually selected; only structures that remained stable after full structural optimization were retained for subsequent calculations of the CO oxidation reaction profiles.

On the basis of the above systematic tests, stable O_sub_ surfaces can only be obtained when two rows of O atoms (eight O atoms in total) are introduced beneath the stepped Rh(332) surface. Under these conditions, two distinct and stable O_sub_ surface structures are identified, denoted as O_sub_ surface1 and O_sub_ surface2, as shown in Figure S7. In O_sub_ surface1, the subsurface O atoms are initially placed at hcp hollow sites in the initial guess and relocate to bridge sites after full structural optimization. In contrast, for O_sub_ surface2, the subsurface O atoms are initially placed at top sites and remain at top sites after optimization. Energetically, O_sub_ surface2 is more stable than O_sub_ surface1 by approximately 0.10 eV. In both O_sub_ surface structures, step1 corresponds to the original step edge present on the clean Rh(332) surface, whose geometric features are modified by the presence of subsurface oxygen. In contrast, step2 emerges from the terrace region as a direct consequence of O_sub_–induced reconstruction. Due to the reconstruction of the surface by underground oxygen, a surface will have two different steps for reaction. Figures S8–S11 show the energetic and geometric information for the reaction pathways on the subsurface-oxygen surfaces. Along these pathways, CO reacts with surface oxygen, while subsurface oxygen acts as a spectator. More detailed transition-state geometries for O_sub_ surface reactions are provided in Table S2.

Under experimental conditions involving subsurface oxygen, the surface can no longer be considered a uniform, well-ordered lattice; rather, multiple local configurations are expected to coexist. Consequently, it is impossible to enumerate all possible subsurface structures. Our objective is therefore to identify thermodynamically stable O_sub_ configurations and to elucidate the mechanistic role of subsurface oxygen. The selected subsurface models are reasonable and representative for this purpose.

Note that regardless of the step, the energy of CO and O co-adsorbed reactants on O_sub_ surface2 is much lower than that on O_sub_ surface1 (0.71 eV and 0.45 eV, respectively). This means that although both structures may exist simultaneously, the reaction pathway on O_sub_ surface2 is far superior to O_sub_ surface1. In O_sub_ surface2, compared to Rh surfaces without subsurface oxygen, the post–transition state bent CO_2_ chemisorption well stabilized relative to desorbed CO_2_. This results in a much deeper chemisorption well for the bent CO_2_ product, and have a larger energy barrier from bending CO_2_ to TS2 than the clean surface, enabling sufficient trapping for thermalization. These findings align with experimental observations, where O_sub_ induces thermal velocity distributions of the desorbing product.


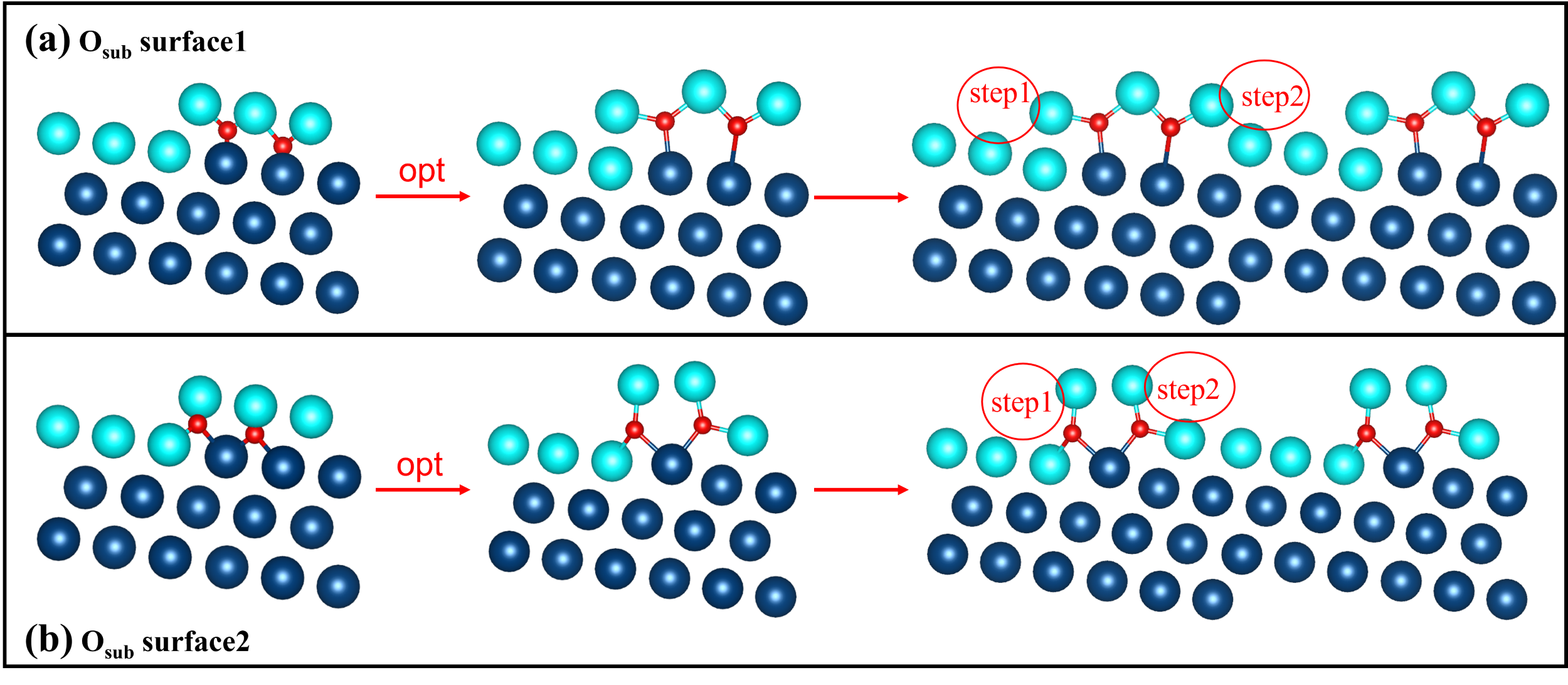


Figure S7. Initial guess (left), optimized structure (middle) and its periodically extended model (right) of the two stable subsurface oxygen surfaces on Rh(332), denoted as (a) O_sub_ surface1 and (b) O_sub_ surface2. The extended models are included to illustrate that step1 is the original step site modified by the subsurface oxygen and step2 emerges from the terrace region as a result of O_sub_–induced reconstruction.


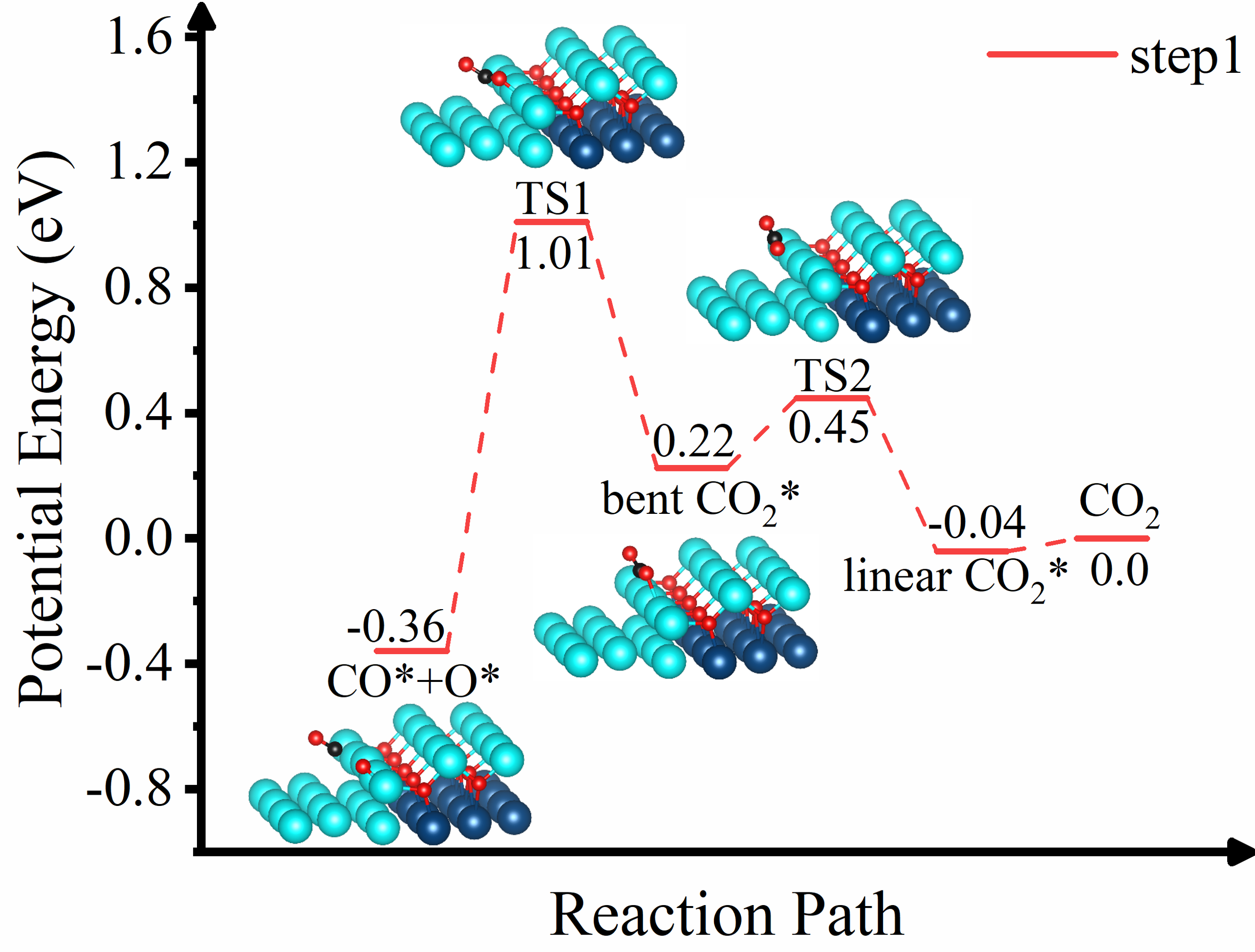


Figure S8. Energetics and geometries of the step1 Path for CO oxidation on O_sub_ surface1.


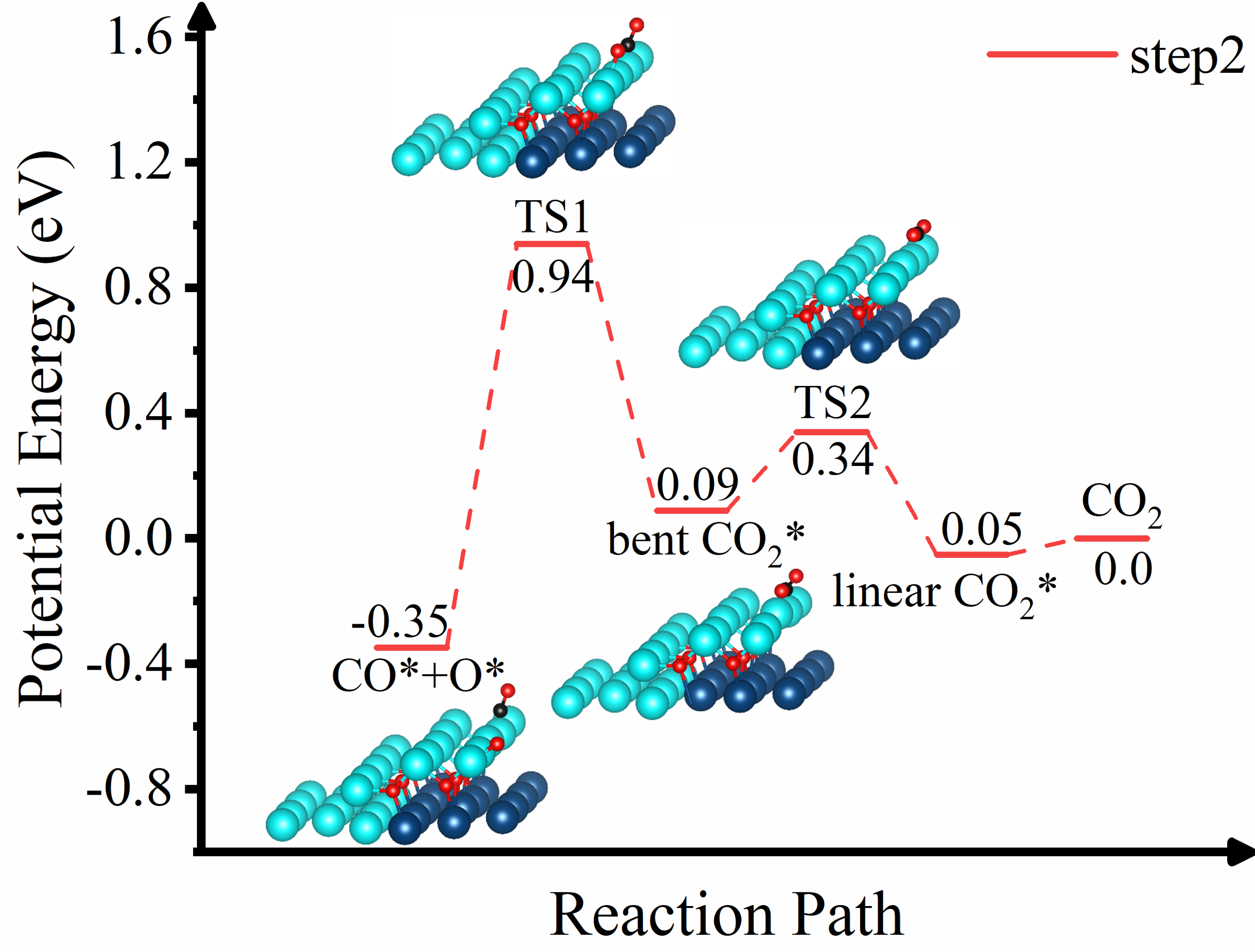


Figure S9. Energetics and geometries of the step2 Path for CO oxidation on O_sub_ surface1.


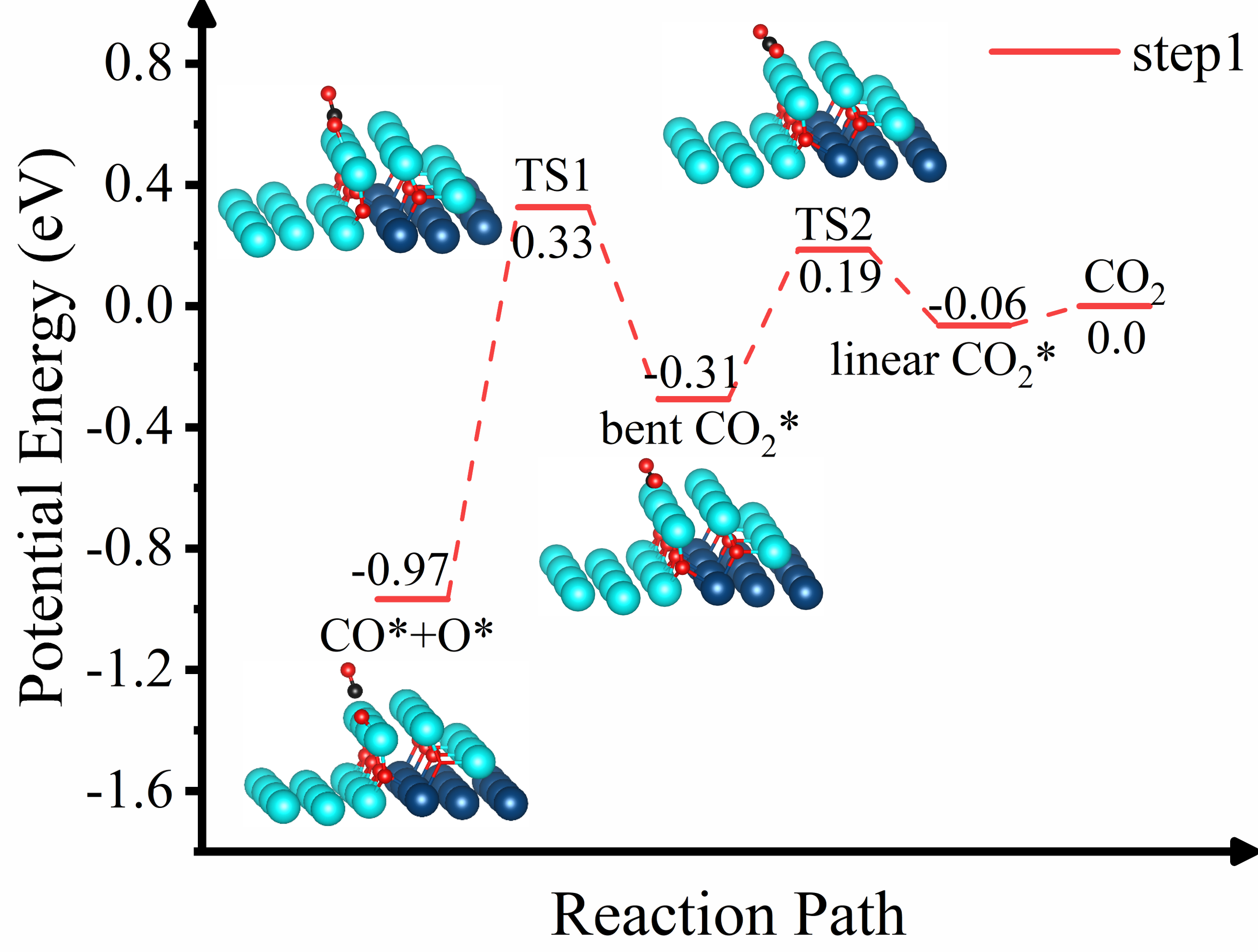


Figure S10. Energetics and geometries of the step1 Path for CO oxidation on O_sub_ surface2.


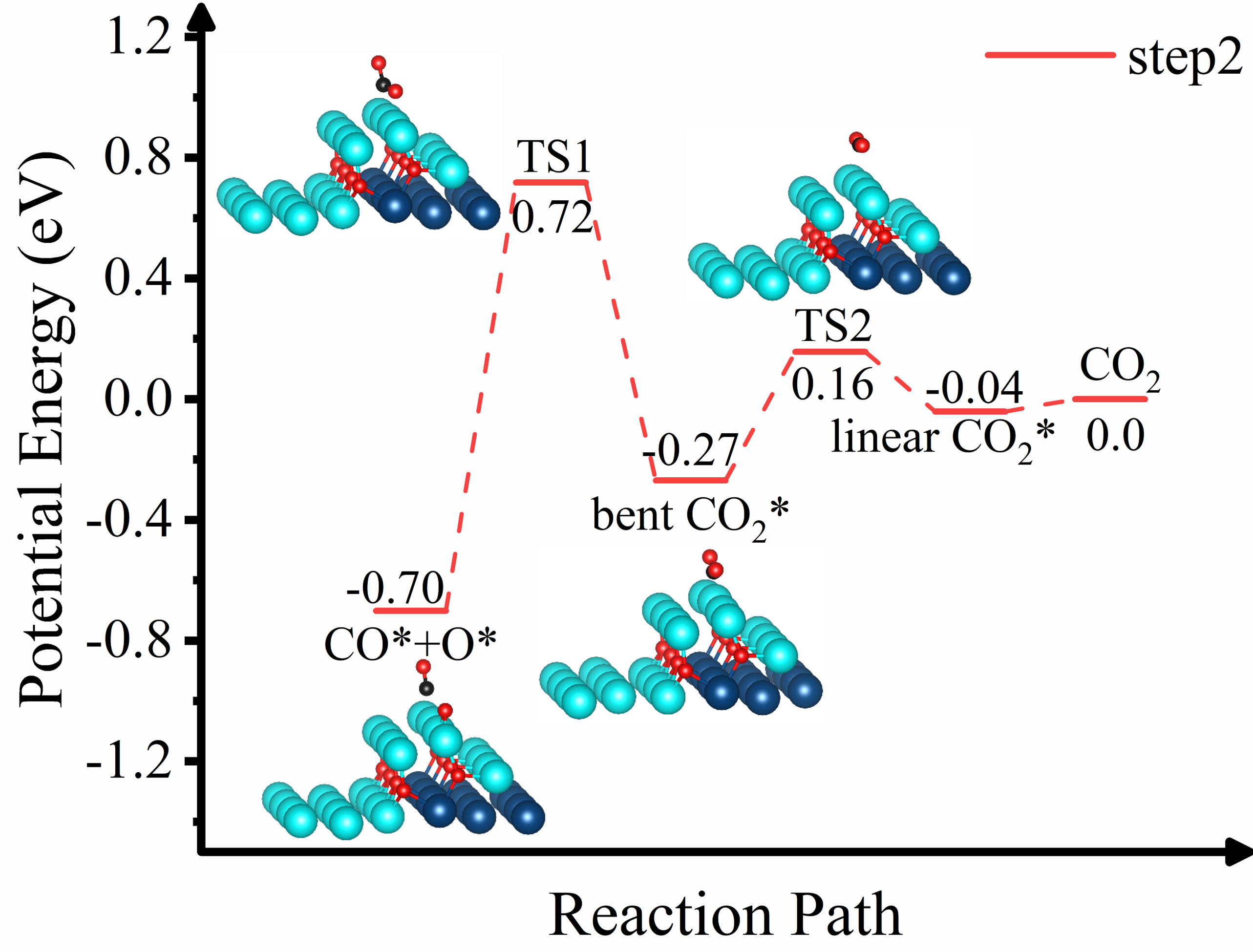


Figure S11. Energetics and geometries of the step2 Path for CO oxidation on O_sub_ surface2.

| Transition state | Reaction Path | *d*_C−Rh_ (Å) | *d*_C−O1_ (Å) | *d*_C−O2_ (Å) | *θ*_OCO_ (deg) |
| --- | --- | --- | --- | --- | --- |
| TS1 | O_sub_ surface1-step1 | 1.98 | 1.83 | 1.18 | 111.65 |
|  | O_sub_ surface1-step2 | 1.97 | 1.82 | 1.18 | 111.48 |
|  | O_sub_ surface2-step1 | 1.96 | 1.83 | 1.18 | 111.70 |
|  | O_sub_ surface2-step2 | 2.00 | 1.84 | 1.18 | 111.46 |
| TS2 | O_sub_ surface1-step1 | 2.66 | 1.21 | 1.19 | 155.97 |
|  | O_sub_ surface1-step2 | 2.59 | 1.20 | 1.20 | 160.90 |
|  | O_sub_ surface2-step1 | 2.84 | 1.20 | 1.18 | 161.30 |
|  | O_sub_ surface2-step2 | 2.81 | 1.19 | 1.19 | 161.48 |

Table S2. Geometric parameters for TS1 and TS2 in different reaction paths in Figure S8-11. *d*_C−Rh_, *d*_C−O1_, and *d*_C−O2_ are the distances of the C−Rh, C−O1, and C−O2 bonds, respectively. *θ*_OCO_ is the OCO bond angle.

**S-III. EXPERIMENTAL DETAILS**

The experiments were conducted on a molecular beam surface-scattering apparatus equipped with two pulsed nozzles whose beams intersect at the surface. A half-section view of the setup is shown in Figure S12. The apparatus has been described previously^.[44, 56]^

**

**

Figure S12. Experimental setup of the molecular beam surface scattering apparatus. For more details, see text.

The source chambers house one molecular beam nozzle each, which are tilted by 20° relative to each other. Hereafter, they are called central (Source 1) and tilted (Source 2) molecular beam. The molecular beam nozzles are home-built and their design bases on a modified Even-Lavie type published in literature.^[43,44]^ The pulsed molecular beams pass through two individually and one jointly differentially pumped chambers before entering the main UHV chamber, ensuring ultra-high vacuum conditions during exposure. During the experiments, pure O₂ (Company) and CO (Company) were introduced through the nozzles, with oxygen supplied via the central nozzle.

The central molecular beam is aligned orthogonally to a laser beam used for ion imaging experiments. The output of a regeneratively amplified femtosecond laser (Spectra-Physics Solstice Ace, <35 fs, 800 nm, 1 kHz) is focused with a 300 mm focal length lens for non-resonant ionization of desorbing surface reaction products. The resulting ions are accelerated by a 2.00 kV repeller toward a phosphor screen (ProxiVision P43, 4.50 kV). Signal amplification is achieved using microchannel plates (MCPs, TOPAG Lasertechnik MCP 56-15). Mass selection is performed by distinguishing ions according to their time-of-flight (TOF): short gated pulses (0.55 kV, 200 ns duration) are applied to the MCPs, and varying the gate delay selects ions of different TOFs. Velocity-map imaging (VMI) conditions are established with an ion lens biased at 1.44 kV. Phosphor screen images are recorded with a CMOS camera (Basler ace acA1920-155um, 1920 × 1200 px). See also Figure 1 in the main article.

The bi-faceted Rh(111)/Rh(332) crystal (MaTecK, ⌀10 mm, 2 mm thickness, 99.99% purity) was mounted on a home-built sample holder using tungsten filaments (⌀0.5 mm), which also enabled resistive heating. The sample temperature was varied between 300 K and 1400 K and monitored by a K-type thermocouple attached directly to the crystal. Surface impurities were removed by repeated cycles of argon sputtering (2.00 kV, <2×10⁻⁷ Torr, 10 µA, 30 min) followed by annealing in an atomic oxygen (AO) atmosphere (1000 K, 1×10⁻⁶ Torr, 20 min) to eliminate carbon contamination. A final flash to 1300 K yielded a clean surface, as verified by Meitner–Auger electron spectroscopy (MAES, Staib Instruments ESA-150) and low-energy electron diffraction (LEED, OCI Instruments 450)

The two crystal facets were distinguished by their vertical position and identified using LEED. The Rh(111) facet exhibited the characteristic (1×1) diffraction pattern, whereas the stepped Rh(332) surface showed split diffraction spots due to terrace-induced reflections. A slit aperture was installed in front of the ion imaging setup to ensure that only desorption products from a selected facet were probed, while those from the other facet were blocked. By adjusting the vertical position of the sample holder, the probed surface could be selectively switched between the two facets.

**S-III. TITRATION EXPERIMENTS**

To estimate the amount of oxygen consumed during a single molecular beam pulse of carbon monoxide, we performed titration experiments. First, we prepared a full monolayer of oxygen at room temperature by dosing the surface for several minutes with molecular oxygen from the O₂ nozzle. This procedure yields a saturated (2×1)-O layer. After the monolayer was established, the O₂ nozzle was turned off and only the CO nozzle remained active. We then monitored the CO₂ reaction product signal as a function of the number of CO beam pulses. Because the O₂ nozzle remained off for the entire measurement, no replenishment of surface oxygen could occur.
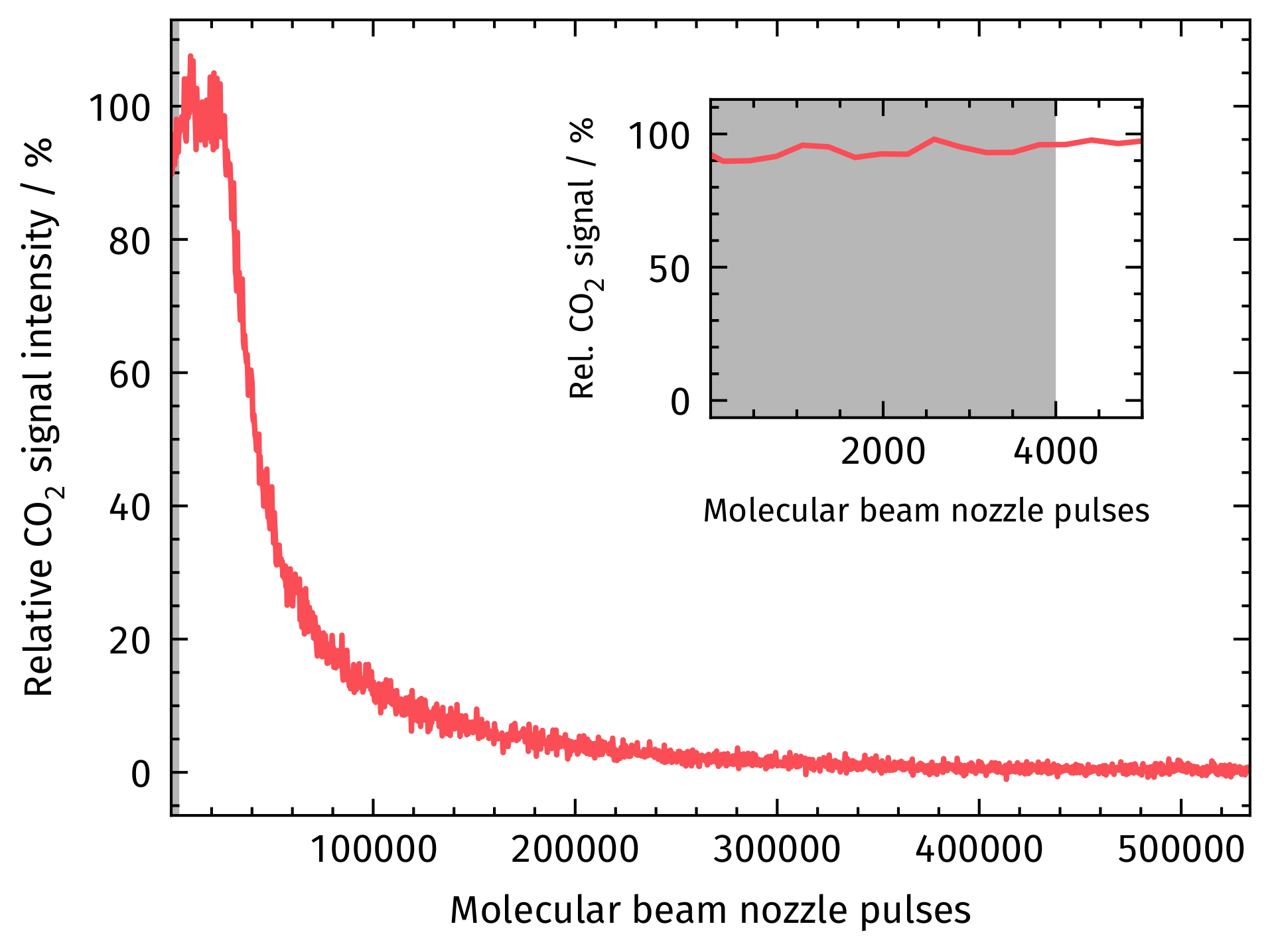


Figure S13. CO_2_ signal intensity as a function of molecular-beam CO pulses. The surface was prepared with a full monolayer of oxygen at room temperature using molecular O_2_, corresponding to a saturated (2×1)-O layer. Only the CO nozzle was operated during the measurement, causing the CO_2_ signal to decrease gradually over time as the oxygen supply was turned off. The nozzle operates at 100 Hz, so the data shown correspond to approximately one hour of measurement. The velocity distributions presented in the main article are averaged over the first 4000 nozzle shots (shaded area).

The results of these titration experiments are shown in Figure S13. The CO₂ signal decreases to half of its initial value after approximately 50 000 shots. For the velocity distributions presented in the main article, we average the images recorded during the first 4 000 shots, as indicated by the shaded region in the figures.

From Figure S13, we determine the oxygen coverage as a function of the number of beam shots. In this analysis, we assume that the total area under the curve in Figure S13 corresponds to a complete monolayer of oxygen. The resulting coverage evolution is shown in Figure S14. During the interval over which we average the velocity distributions (grey shaded region), the coverage decreases from 100 % to approximately 92 %. We do not expect this modest change to significantly affect the measured velocity distributions. Consistent with this, we have always observed hyperthermal velocity distributions even at lower coverages, as long as no subsurface oxygen states were involved.


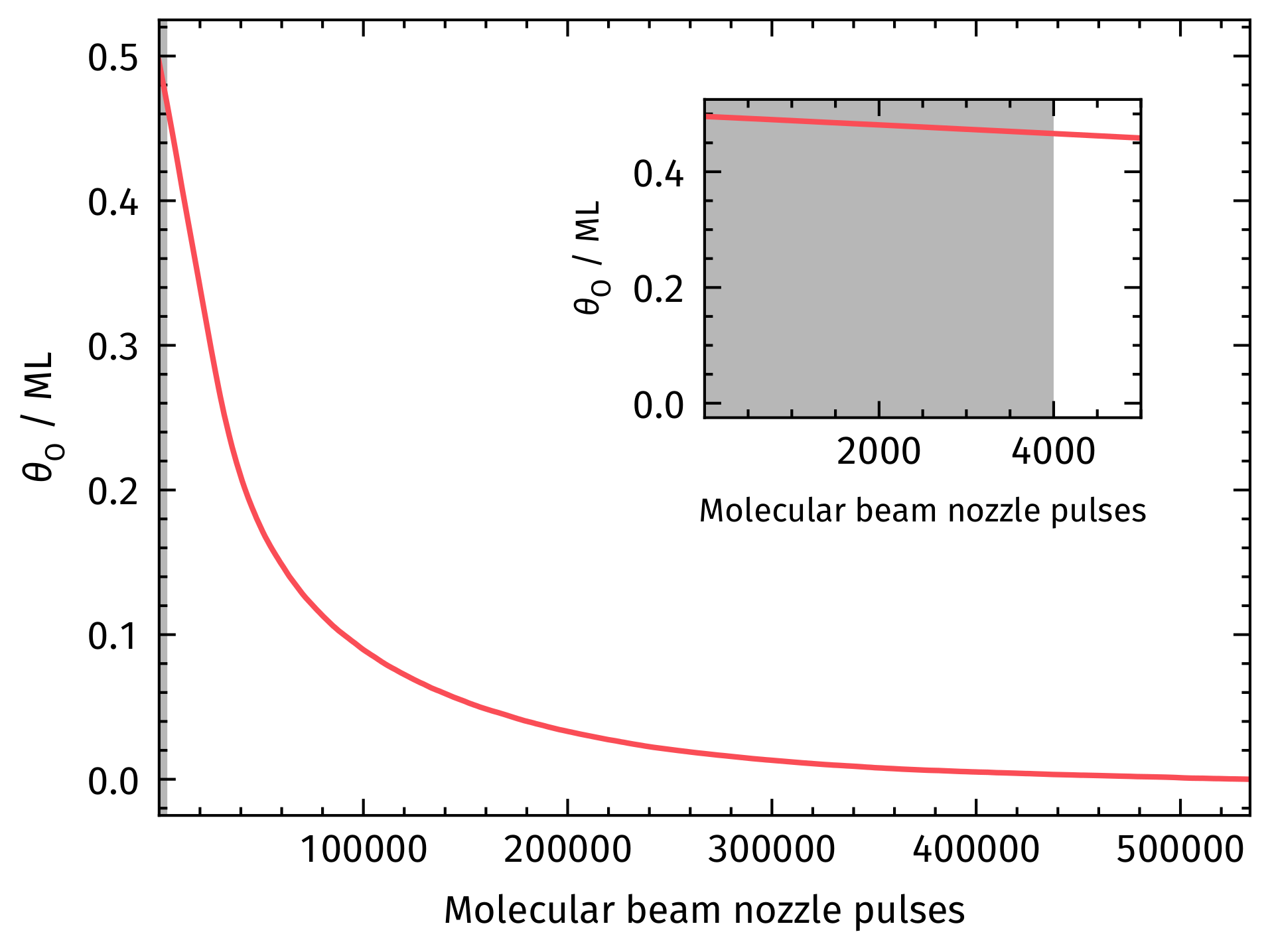


Figure S14. Oxygen coverage as a function of molecular-beam CO pulses. The surface was prepared with a full monolayer of oxygen at room temperature using molecular O_2_, corresponding to a saturated (2×1)-O layer. Only the CO nozzle was operated during the measurement, causing the CO_2_ signal to decrease gradually over time as the oxygen supply was turned off. The nozzle operates at 100 Hz, so the data shown correspond to approximately one hour of measurement. The velocity distributions presented in the main article are averaged over the first 4000 nozzle shots (shaded area).

References

[85] L. Zhou, A. Kandratsenka, C. T. Campbell, A. M. Wodtke, H. Guo, Origin of thermal and hyperthermal CO_2_ from CO oxidation on Pt surfaces: The role of post-transition-state dynamics, active sites, and chemisorbed CO_2_*, Angew. Chem. Int. Ed.,* **2019**, *58*, 6916-6920.

[86] G. Henkelman, H. Jónsson, A dimer method for finding saddle points on high dimensional potential surfaces using only first derivatives*, J. Chem. Phys.,* **1999**, *111*, 7010-7022.

[87] G. Henkelman, B. P. Uberuaga, H. Jónsson, A climbing image nudged elastic band method for finding saddle points and minimum energy paths*, J. Chem. Phys.,* **2000**, *113*, 9901-9904.

[88] P. E. Blöchl, Projector augmented-wave method*, Phys. Rev. B,* **1994**, *50*, 17953-17979.

[89] G. Kresse, D. Joubert, From ultrasoft pseudopotentials to the projector augmented-wave method*, Phys. Rev. B,* **1999**, *59*, 1758-1775.

[90] J. P. Perdew, K. Burke, M. Ernzerhof, Generalized gradient approximation made simple*, Phys. Rev. Lett.,* **1996**, *77*, 3865-3868.

[91] G. Kresse, J. Furthmuller, Efficient iterative schemes for ab initio total-energy calculations using plane wave basis set*, Phys. Rev. B,* **1996**, *54*, 11169-11186.

[92] G. Kresse, J. Furthmuller, Efficiency of ab initio total energy calculations for metals and semiconductors using plane wave basis set*, Comp. Mater. Sci.,* **1996**, *6*, 15-50.

[93] U. Even, J. Jortner, D. Noy, N. Lavie, C. Cossart-Magos, Cooling of large molecules below 1 K and He clusters formation*, The Journal of Chemical Physics,* **2000**, *112*, 8068-8071.

[94] G. B. Park, B. C. Krüger, S. Meyer, D. Schwarzer, T. Schäfer, The ν6 fundamental frequency of the A ̃ state of formaldehyde and Coriolis perturbations in the 3ν4 level*, The Journal of Chemical Physics,* **2016**, *144*.
